# Tetraspanin profiles of serum extracellular vesicles reflect functional limitations and pain perception in knee osteoarthritis

**DOI:** 10.1186/s13075-023-03234-0

**Published:** 2024-01-22

**Authors:** Anne-Mari Mustonen, Mari Palviainen, Laura Säisänen, Lauri Karttunen, Sylvain Tollis, Amir Esrafilian, Jusa Reijonen, Petro Julkunen, Pia R-M Siljander, Heikki Kröger, Jussi Mäki, Jari Arokoski, Petteri Nieminen

**Affiliations:** 1 Institute of Biomedicine, School of Medicine, Faculty of Health Sciences, University of Eastern Finland, Kuopio, Finland; 2 Department of Environmental and Biological Sciences, Faculty of Science, Forestry and Technology, University of Eastern Finland, Joensuu, Finland; 3 EV core and EV group, Molecular and Integrative Biosciences Research Programme, Faculty of Biological and Environmental Sciences University of Helsinki, Helsinki, Finland; 4 Department of Clinical Neurophysiology, Kuopio University Hospital, Kuopio, Finland; 5 Department of Technical Physics, Faculty of Science, Forestry and Technology, University of Eastern Finland, Kuopio, Finland; 6 Department of Physical and Rehabilitation Medicine, Central Finland Hospital Nova, Jyväskylä, Finland; 7 Department of Orthopaedics, Traumatology and Hand Surgery, Kuopio University Hospital, Kuopio, Finland; 8 Kuopio Musculoskeletal Research Unit, University of Eastern Finland, Kuopio, Finland; 9 Department of Rehabilitation, Kuopio University Hospital, Kuopio, Finland; 10 Department of Physical and Rehabilitation Medicine, Helsinki University Hospital, Helsinki, Finland and University of Helsinki, Helsinki, Finland

**Keywords:** Cluster of differentiation, Exosome, Extracellular vesicle, Functionality, Knee, Osteoarthritis, Pain, Platelet, Tetraspanin, Transcranial magnetic stimulation

## Abstract

**Background:**

Emerging evidence suggests that extracellular vesicles (EVs) can play roles in inflammatory processes and joint degradation in primary osteoarthritis (OA), a common age-associated joint disease. EV subpopulations express tetraspanins and platelet markers that may reflect OA pathogenesis. The present study investigated the associations between these EV surface markers and articular cartilage degradation, subjectively and objectively assessed pain, and functional limitations in primary knee OA (KOA).

**Methods:**

Serum EVs were determined by high-sensitivity flow cytometry (large CD61^+^ EVs) and single particle interferometric reflectance imaging sensor (small CD41^+^, CD63^+^, CD81^+^, and CD9^+^ EVs) from end-stage KOA patients and controls (*n* = 8 per group). Knee pain and physical functions were assessed with several health- and pain-related questionnaires, established measurements of physical medicine, and neuromuscular examination. The obtained data were analyzed using supervised and unsupervised univariate and multivariate models.

**Results:**

With the combined dataset of cartilage thickness, knee function, pain, sensation, and EV molecular signatures, we identified highly correlated groups of variables and found several EV markers that were statistically significant predictors of pain, physical limitations, and other aspects of well-being for KOA patients, for instance CD41^+^/CD63^+^/CD9^+^ small EVs associated with the range of motion of the knee, physical performance, and pain sensitivity.

**Conclusions:**

Particular serum EV subpopulations showed clear associations with KOA pain and functional limitations, suggesting that their implications in OA pathophysiology warrant further study.

**Supplementary Information:**

The online version contains supplementary material available at 10.1186/s13075-023-03234-0.

## Background

Chronic pain conditions, such as osteoarthritis (OA), rheumatoid arthritis (RA), and fibromyalgia, constitute a significant public health problem [1]. The molecular mechanisms that underlie the pathogeneses of these conditions remain inadequately understood, and there is an urgent need for biomarkers for clinical use in conditions characterized by chronic pain as well as in the overlapping pain categories, including nociceptive, neuropathic, and inflammatory pain. Extracellular vesicles (EVs) would be of special interest, as not only do they function in intercellular communication but they are also involved in pain processes and have the ability to ameliorate pain and to transport specific cargo molecules with great potential as pain biomarkers [2].

The current understanding of the etiology of OA pain remains inconclusive [3], and, at least partly because of this, pain management for knee OA (KOA) is unspecific. OA pain is complex and multimodal, and its severity and location can differ between individuals and change over the course of the disease [4]. Structural features, including cartilage degradation, subchondral bone remodeling, osteophyte development, and synovitis, may all contribute to the generation and maintenance of pain, but OA can also be a mixed pain state combining peripheral nociception and centralized pain modulation [3]. Pain medications for OA include oral (and topical) non-steroidal anti-inflammatory drugs, paracetamol, and opioids that often have limited beneficial influence and/or adverse side effects [5]. Knee pain experienced by the patient is not necessarily linked to the radiological degree of joint damage [6], and it would be important to unveil biochemical signatures correlating with KOA pain, functional limitations, and neuromuscular function.

Previous studies have demonstrated alterations in the levels and profiles of EVs in body fluids in chronic pain conditions, including joint diseases [7, 8]. For instance, patients with end-stage KOA had higher counts of exosomes (EXOs, a subpopulation of EVs) in synovial fluid (SF) and higher cytokine and chemokine levels in these EXOs compared to patients with early KOA [9]. EVs have been proposed to stimulate pro-inflammatory pathways by transporting inflammatory factors and cartilage-degrading proteinases and by inducing their production by synovial joint cells [10], and plasma EVs with tumor necrosis factor *α* could be an independent predictor of KOA progression [11]. Plasma EV miRNAs were suggested to be important in nociception and to serve as biomarkers for chronic neuropathic pain [12]. Unfortunately, previous studies did not usually compare the determined EV profiles to functional limitations or to the intensity of pain experienced by the patients or model animals. In rodents, mesenchymal stem cell (MSC)-derived EVs have been demonstrated to reduce OA-related pain behavior [13–15]. In humans, it is also known that MSCs can relieve pain, while data on similar effects by EVs remain scarce [16].

Currently, there are no established biochemical or genetic markers that would reliably identify early OA or predict OA progression and, for instance, cartilage-derived biomarkers are of limited clinical utility [17]. Several molecular biomarkers, such as cytokines, neoepitopes, adhesion molecules, and growth factors, have been associated with pain severity or joint degradation, but they do not show specificity for OA pain [3, 4]. Amino acids, sugars, and related metabolites also belong to the potential biomarkers found in OA SF [18], but none of these are actively being used in clinical medicine. As blood is the most routinely collected biofluid, screening for biomarkers from plasma/serum would appear as the most practical approach. In addition to non-invasive biomarkers to predict OA, EV profiling could also provide potential therapeutic targets for both pain and OA pathogenesis, thereby facilitating the design of personalized pain management strategies. In fact, blood-derived EVs can function as regulators of cartilage extracellular matrix metabolism and be candidates for new cell-free therapeutic approaches for OA [19].

Although EVs present a novel approach in biomarker research, the lack of standardized preanalytical and analytical methods hampers breakthroughs in this field. Since EVs are small and heterogeneous in their attributes, they are difficult to analyze, e.g., with conventional flow cytometry [20]. Moreover, classifying EVs according to biogenesis pathways remains difficult, and, for this reason, they have been recommended to be categorized based on physical characteristics, such as size, to “small EVs” (sEVs) and “large EVs” (lEVs) by the MISEV2018 guidelines [21]. Here, we used two complementary EV methods capable of single EV detection: (*i*) high-sensitive flow cytometry with light scatter calibration based on the Mie theory allowing to accurately size lEVs (200–1000 nm in diameter) [22] and (*ii*) single particle interferometric reflectance imaging sensor (SP-IRIS) allowing the single detection of sEVs (50–200 nm) [23].

The present study focused on EVs carrying proteins of the tetraspanin superfamily, which are among the most abundant membrane proteins in EVs [24]. Serum samples contain high concentrations of platelet-derived EVs, and, of the selected markers, CD41 and CD61 are used to detect platelet-derived EVs [25], while classical tetraspanins, CD9, CD63, and CD81, are utilized as universal EV markers [24]. Tetraspanins have the capacity to interact with several receptors and signaling molecules at the membrane, organizing specialized tetraspanin-enriched microdomains with possible roles in the EV biogenesis, selection of EV cargo, binding and uptake of EVs by target cells, and the ability of EVs to present antigens during immune response. The roles of tetraspanins in arthritis remain unresolved, but their profiles have been studied in the plasma EVs of RA patients, who had higher proportions of single-positive CD81 and CD9 sEVs but lower levels of double-positive CD81/CD9 sEVs than healthy controls [26]. It has also been documented that the proportions of annexin V^+^/CD41a^+^ EVs increase in plasma with disease activity of RA and systemic lupus erythematosus that are both autoimmune-driven inflammatory disorders [27], and that CD9 and CD81 can participate in OA and RA development in animal models [28, 29].

The present study characterized tetraspanin profiles in the serum EVs of controls and KOA patients and compared them to radiological and physical findings related to functionality and pain perception in the same subjects. The aims were to find new biological markers for KOA that would correlate to (*i*) the degradation of articular cartilage, (*ii*) subjectively assessed joint pain and function, and (*iii*) objectively assessed joint pain, sensation, and function by using several health- and pain-related questionnaires, established tools of physiatry, and neuromuscular examination with transcranial magnetic stimulation (TMS). We hypothesized that (*i*) the presence of KOA and the severity of its symptoms would associate with changes in serum EV subpopulations compared to the controls and that (*ii*) from the initially wide range of subjective and objective physical and pain-related data, it would be possible to detect the strongest correlates between EVs and KOA variables with statistical and bioinformatic analyses. The strongest correlates could become candidates for further translational studies on KOA pathogenesis, early detection, and ultimately treatment.

## Materials and methods

### Ethics, subjects, and sampling

The study protocol was approved by the Ethical Committee of the Kuopio University Hospital (#140/2017) in accordance with the Helsinki Declaration, and all subjects provided written informed consent to donate their blood samples for research purposes. A total of 8 patients (2 men, 6 women) with end-stage primary KOA who underwent total knee arthroplasty and 8 healthy volunteers (5 men, 3 women) with no clinical history of joint diseases were recruited for the study (Table 1). The KOA patients were operated at the Kuopio University Hospital in 2020–2022. Inclusion criteria were as follows: 45–70 years of age; referral to total knee arthroplasty; radiographically defined moderate to severe KOA; tibiofemoral joint pain during most days, relatively normal range of motion (ROM), and no clinical instability of the knee. Exclusion criteria were as follows: severe pain, limited ROM, or substantial instability of the knee caused by other diseases; radiologically too mild or too advanced KOA; neurological or metabolic diseases, active malignancies, inflammatory arthritis, earlier restorative surgery of the knee; metal objects or implants in the body (if not compatible with magnetic resonance imaging [MRI] scanners), cardiac pacemaker; body mass index (BMI) > 33 kg/m^2^; and thigh circumference > 52 cm (measured 12 cm proximal from the lower margin of the patella). Age, gender, body weight, height, BMI, and medication were recorded. There were no differences in the sex ratios or average body masses between the study groups, but the KOA patients were older and had a higher average BMI than the controls (Table 1).

**Table 1:** General characteristics of the patients (mean ± SE)

Group	KOA	Control	*p*
Sex (M/F)	2/6	5/3	0.315
Age	64 ± 2	29 ± 2	< 0.001
Body mass	90.9 ± 5.05	77.9 ± 3.29	0.105
BMI	32.2 ± 0.41	25.4 ± 0.91	< 0.001

*KOA*, knee osteoarthritis; *M*, male; *F*, female; *BMI*, body mass index, sex ratios were tested with the Fisherʼs exact test, the other comparisons with the Mann–Whitney *U* test

Venous blood was collected after overnight fasting from the controls and from the KOA patients before joint replacement surgery using BD Vacutainer® Clot Activator tubes (BD, Belliver Industrial Estate, Plymouth, UK). After a 30-min incubation at room temperature (RT) to allow clotting, the samples were centrifuged at 2500 × *g* for 15 min at RT. The supernatant was subsequently transferred to a new tube and centrifuged at 2500 × *g* for 15 min at RT [25]. The obtained serum was aliquoted and stored at − 80 °C.

### Measurements of physical function, pain, and cartilage thickness

Physical measurements are described in more detail in Supplementary Material S1. The ROM (flexion, extension) of each control, contralateral (CL), and OA knee joint was measured with standard goniometry [30]. Physical function measurements included a 30-s chair-stand test, a 4 × 10 m fast-paced walk test, and a 12-step stair-climb test recommended by the Osteoarthritis Research Society International [31]. Pain and sensation were evaluated with visual analog scale (VAS) [32], pressure pain (PPT), thermal detection, and heat pain thresholds [33–35], and two-point discrimination (TPD) [36].

Articular cartilage thicknesses in medial and lateral tibia and femur of each control, CL, and OA knee were measured based on MRI (Philips Achieva 3.0 T X, Philips, Eindhoven, the Netherlands; or Siemens MAGNETOM Vida, Siemens Healthcare, Erlangen, Germany). Cartilage thicknesses were calculated through an automated pipeline implemented in Python based on the tissue geometries obtained from a deep-learning segmentation tool, nnU-Net [37] (Supplementary Material S2). The selected regions were located in the middle and around the principal load-bearing areas that are the most susceptible to cartilage degradation (Supplementary Fig. S1).

### Neuromuscular examination

TMS was performed using a navigated TMS system (NBS 4.3 research version, Nexstim, Helsinki, Finland) with a figure-of-eight coil producing pulses with a biphasic waveform. The electromyogram was measured in a bipolar montage using self-adhesive electrodes on the *tibialis anterior* (TA) muscle and reference electrodes on the tibial bone. Both hemispheres were studied in a randomized order.

First, a coarse mapping was performed using stimulus intensity producing motor evoked potential (MEP) amplitudes of approximately 0.5–1.0 mV to find the hotspot, i.e., the cortical site producing the highest amplitude MEPs repeatedly. At this target, the coil was rotated in approximately 10-degree steps to find the optimal orientation [38]. Thereafter, the resting motor threshold (rMT) was defined using the system-integrated adaptive threshold hunting paradigm [39, 40] or, if the rMT exceeded 80% of the maximum stimulator output, the adaptive threshold hunting tool with a corresponding function [41].

Motor mapping was performed at a stimulus intensity of 105% of rMT [42] with an approximately constant coil orientation. A rectangular grid was visualized on the cortical view of the navigated TMS system, and one stimulus was applied per grid square (5 mm between grid nodes), using an interstimulus-interval of 4–6 s until there was a rim of negative responses [43]. The representation area (Map TA) was calculated using the spline interpolation method [44]. Twenty trials of long-interval cortical inhibition (LICI) were assessed at the hotspot with the interstimulus interval of 100 ms, both pulses applied at suprathreshold stimulus intensity (120% rMT). LICI was determined as the mean of the second divided by the mean of the first MEP amplitude (%).

### Self-reported questionnaires

In addition to medial cartilage thicknesses and physiatric and neuromuscular data collected preoperatively during the outpatient visits, the EV results were correlated to data from several health- and pain-related questionnaires. They included painDETECT [45], Western Ontario and McMaster Universities Osteoarthritis Index (WOMAC) [46], RAND-36 measure of health-related quality of life [47], Beck Depression Inventory (BDI), Beck Anxiety Inventory (BAI) [48], and Pain Self-Efficacy Questionnaire (PSEQ) [49]. These questionnaires were not obtained from the controls.

### Analyses of EVs

The concentration of lEVs derived from platelets was measured by high-sensitive flow cytometry (A50 Micro, Apogee Flow Systems, Hemel Hempstead, UK). Counts of EVs are reported per mL of serum (*n* = 8 per group), including particles (*i*) with a diameter between 200 and 1000 nm according to the applied flow cytometry scatter ratio (10) and (*ii*) positive for the fluorescent surface markers used. Prior labeling, serum samples were pre-diluted in 0.1 μm pre-filtered 10 mM Hepes − 140 mM NaCl, pH 7.2 (NH buffer) to obtain event rates between 2000 and 3000/s to prevent swarm detection when triggering on side scatter [50]. The pre-dilution varied per serum sample and ranged from 2-fold to 50-fold. The pre-diluted sample (20 μl) was incubated with 1.75 μl of CD61-APC (allophycocyanin, clone VI-PL2, #564174, BD Biosciences, San Jose, CA, USA) or isotype control (APC IgG1 Kappa, #567155, BD Biosciences) and kept in the dark at RT for 2 h. After the incubation, 200 μl of NH buffer was added, and the samples were measured. Rosetta Calibration (Exometry, Amsterdam, the Netherlands) was used to calibrate side scatter related to the diameter of EVs in nm and ApogeeMix (Apogee Flow Systems) for flow rate quantification. For the improved reproducibility of the analysis, we followed the guidelines of the standardized MIFlowCyt-EV framework [51]. The data were analyzed using FLowJo (*v*10, FlowJo, Ashland, OR, USA). Details of the experiment, including representative scatter plots and gating strategy, are available in Supplementary Fig. S2.

The serum-derived sEVs were analyzed with SP-IRIS using the ExoView™ Plasma Tetraspanin kit and the ExoView™ R100 scanner (NanoView Biosciences, Unchained Labs, Pleasanton, CA, USA) according to the manufacturer’s instructions. The serum samples (*n* = 8 per group) were diluted with the incubation buffer provided in the kit at optimized manner based on nanoparticle tracking analysis (NanoSight instrument LM14, Malvern Panalytical, Malvern, UK). For nanoparticle tracking analysis, the serum samples were pre-diluted in 0.1 μm pre-filtered DPBS to obtain 40–100 particles/frame, and five 30-s videos were recorded with camera level 14. The data were analyzed using the NTA 3.0 software (Malvern Panalytical) with the detection threshold of 4 and the screen gain of 10. The pre-dilution varied per serum sample and ranged from 70-fold to 250-fold. The diluted samples were added directly to the ExoView chips and incubated at RT for 16 h. The chips were then stained using fluorescently labeled antibodies (CD9/CD63/CD81, provided in the kit), washed, dried, and scanned. The obtained data were analyzed using the NanoViewer analysis software *v*3.0 (NanoView Biosciences, Unchained Labs) with sizing thresholds set to 50–200 nm diameter.

### Statistics and bioinformatics

#### Conventional statistical analyses

Statistical analyses of measured variables across samples were conducted with the IBM SPSS *v*27 software (IBM, Armonk, NY, USA). Comparisons between control and OA were performed with the Mann–Whitney *U* test and those between control, CL, and OA with the Kruskal–Wallis one-way analysis of variance. For controls, the average value of the right and left knee was calculated and used for the analyses. Sex ratios were compared with the Fisherʼs exact test. To assess the potential effects of confounding factors, comparisons were also performed with the generalized linear model (GLM) using age and BMI as covariates. The *p*-value < 0.05 was considered statistically significant. The *p*-values of comparisons across groups were adjusted for multiple hypothesis testing controlling the false discovery rate by using the Benjamini–Hochberg procedure (critical value = 0.00658). The results are presented as the mean ± standard error (SE).

#### Unsupervised analyses: principal component analysis (PCA) and hierarchical clustering (HC)

All variables were first converted to Z-scores in IBM SPSS *v*27 using mean and variance across study subjects as parameters for the normalization. *Z*-score data were transferred to ClustVis (https://biit.cs.ut.ee/clustvis/) where PCA and HC analyses were conducted. Categorical variables (group and sex) were excluded from these analyses. We used correlations as the clustering distance and the Ward (unsquared distances) clustering method [52] for HC.

#### Correlation analysis and group identification

**Fig. 1:**
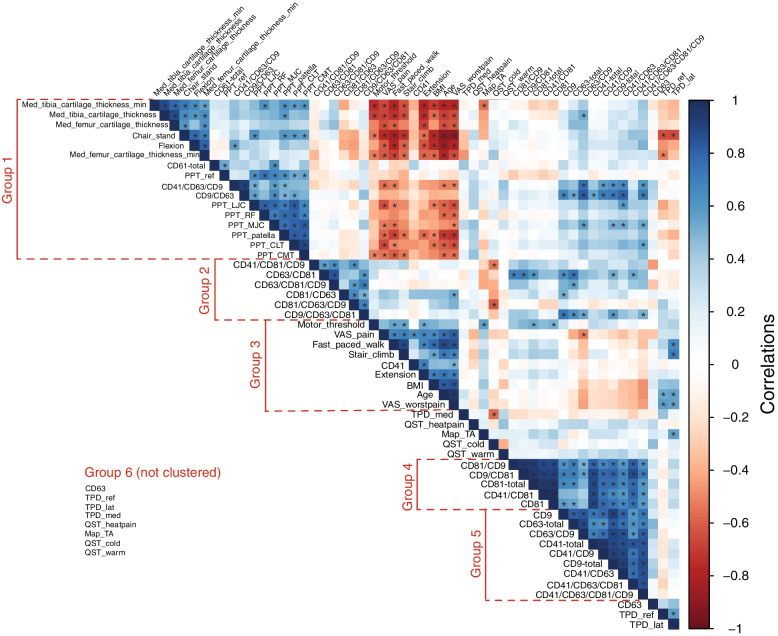
Medial cartilage thickness, physical function, pain, and serum extracellular vesicle variables cluster in highly correlated groups. Pairwise Pearson correlation coefficients between variables, computed across all subjects and color-coded as indicated. Dark blue (respectively red) on the correlogram indicates strong positive (respectively negative) correlations in variables’ *Z*-scores. Asterisks indicate statistically significant correlations (*p* < 0.05)

We used R *v*4.1.2 [53] and its corrplot library to compute Pearson (linear) correlations between the variables and to cluster the variables according to these correlations. This analysis identified 5 distinct clusters of variables, to which we refer as groups 1–5, while the 8 variables that associated less with the clusters were gathered into group 6 (Fig. 1).

#### Random forest (RF) analysis, data enrichment, and feature importance quantification

RF analysis was performed using the sklearn Python package, following our approach published in [54]. To build the RF classifier, we used the following parameters: n_estimators = 100, criterion = ‘entropy’, max_depth = None, min_samples_split = 2, min_samples_leaf = 1, min_weight_fraction_leaf = 0.0, max_features = 6, max_leaf_nodes = None, min_impurity_decrease = 0.0, bootstrap = True, oob_score = False, n_jobs =  − 1, random_state = None, verbose = 0, warm_start = False, class_weight = None, ccp_alpha = 0.0, max_samples = None. The analysis script is provided as an annotated Jupyter notebook (Supplementary Material S3).

As detailed in [54], we took advantage of the strong intra-group correlations between the different variables to enrich the original dataset 1000-fold, by sampling each group only once and by repeating the operation 1000 times until we obtained a reduced dataset of 6 features only (number of groups) but encompassing 1000 more pseudo-subjects (hereafter referred to as the 1000 × dataset). We next verified that the information encoded in the 6-features data was equivalent to that of the full dataset by randomly splitting the enriched 1000 × dataset in training (80% of the pseudo-subjects) and testing data (20%). This way, both training and testing datasets included 6-features sub-samplings of *all* the original subjects. The RF classifier trained with this training dataset accurately predicted the diagnosis of ~ 99.9% of the test pseudo-subjects. Hence, sampling one variable per group for model training was sufficient to predict the subject type (control, OA) when given another variable, demonstrating the redundancy of information within each group of variables. To test the power of the approach to predict new, uncategorized subjects, we split the training and testing datasets *before* data enrichment (performed as described above).

We estimated the relevance of individual variables in distinguishing control vs. OA pseudo-subjects by computing the feature_importances_ function on the training data in sklearn. We emphasize that sklearn’s feature_importances_ function is based on the mean decrease in impurity in the RF model and, therefore, is only reliable when model predictions are accurate.

## Results

### Cartilage thicknesses, physical and neuromuscular determinations

The OA knees had significantly lower articular cartilage thicknesses in medial tibia and medial femur load-bearing regions, while the values of the CL knees were in between the control and OA knees (Supplementary Table S1). There were no significant differences in the cartilage thicknesses of lateral tibia or femur between the control, CL, or OA knees. Flexion and extension (degrees) were more limited in the OA knees compared to the CL knees and the knees of the controls (Supplementary Table S2). Chair-stand, fast-paced walk, and stair-climb tests indicated impaired functionality due to KOA, supported by the self-reported physical function scores from the WOMAC questionnaire (Supplementary Table S3). Regarding the above-mentioned variables, group differences in minimum cartilage thickness in medial tibia, flexion, extension, chair-stand, fast-paced walk, and stair-climb tests remained significant after the Benjamini–Hochberg procedure.

TPD did not differ between the control, CL, and OA knees on the lateral or medial sides, but it was increased by KOA in the non-dominant upper extremity (Supplementary Table S4). PPT was lower in the patella, lateral joint capsule, and lateral and medial tibial condyles of the affected joint compared to the control knees, and it was also lower in the medial tibial condyle of the CL knee compared to the controls. There were no differences in the thresholds for the detection of cold, warm, or heat pain between the joint groups. Compared to the control subjects, the rMT values were elevated in the KOA patients on both the OA and CL sides, while Map TA and LICI remained unaffected by KOA. The current and worst VAS pain were higher for the OA knees than the control and CL knees (Supplementary Table S5), and, of the above-mentioned variables, the differences in rMT and in current and worst VAS pain remained significant after the Benjamini–Hochberg correction.

### Total counts of EVs and co-localization of EV surface markers

Flow cytometry detected an average concentration of 8.0 × 10^5^ CD61^+^ lEVs per ml of human serum with diameters of 200–1000 nm (Supplementary Fig. S3A). With SP-IRIS, CD41^+^ EVs were the most abundant among the examined sEVs in serum samples, followed by CD63^+^, CD81^+^, and CD9^+^ EVs (Supplementary Fig. S3B). These particles were 50–200 nm in diameter, and their concentrations ranged between 12.1 and 18.7 × 10^6^ EVs/ml. There were no differences in the total counts of CD61^+^, CD41^+^, CD63^+^, CD81^+^, or CD9^+^ EVs between the study groups.

Among the sEVs measured by SP-IRIS, CD41 was mostly co-localized with CD9 (26%) or CD81 (25%), or it was triple-positive with CD63 and CD9 (26%) (Supplementary Fig. S4). KOA did not affect the counts or % of CD41^+^ EV subpopulations (Supplementary Fig. S4, S5A). Approximately 42% of CD63 did not co-localize with other studied tetraspanins, 32% co-localized with CD9, and 19% with CD81 (Supplementary Fig. S4, S5B). The % of CD63^+^/CD9^+^ EVs was lower in KOA samples compared to serum from the controls (Mann–Whitney *U* test, *p* = 0.003). Regarding CD81, 38% of the EVs were single-positive, and 30% double-positive for CD81 and CD9 (Supplementary Fig. S4, S5C). KOA serum contained more CD81^+^/CD63^+^ and CD81^+^/CD63^+^/CD9^+^ EVs than control serum (Mann–Whitney *U* test, *p* = 0.021–0.027). Finally, CD9 was mainly co-localized with CD81 (33%), CD63 (29%), or did not co-localize with other measured tetraspanins (28%) (Supplementary Fig. S4, S5D). There were no differences in the counts or % of these EV subpopulations between the study groups. After controlling for multiple hypothesis testing, the observed differences in CD81^+^/CD63^+^ and CD81^+^/CD63^+^/CD9^+^ EVs did not remain significant.

### Associations of EVs to knee function, sensation, and pain

To quantify how EV subpopulations associate with knee functionality and pain parameters, we performed a systematic analysis of pairwise (Pearson) correlations between all variables, across the controls and KOA patients. This analysis identified 5 groups of variables with strong intra-group correlations (Fig. 1). Despite the small number of subjects, many of these correlations were statistically significant (*p* < 0.05).

**Fig. 2:**
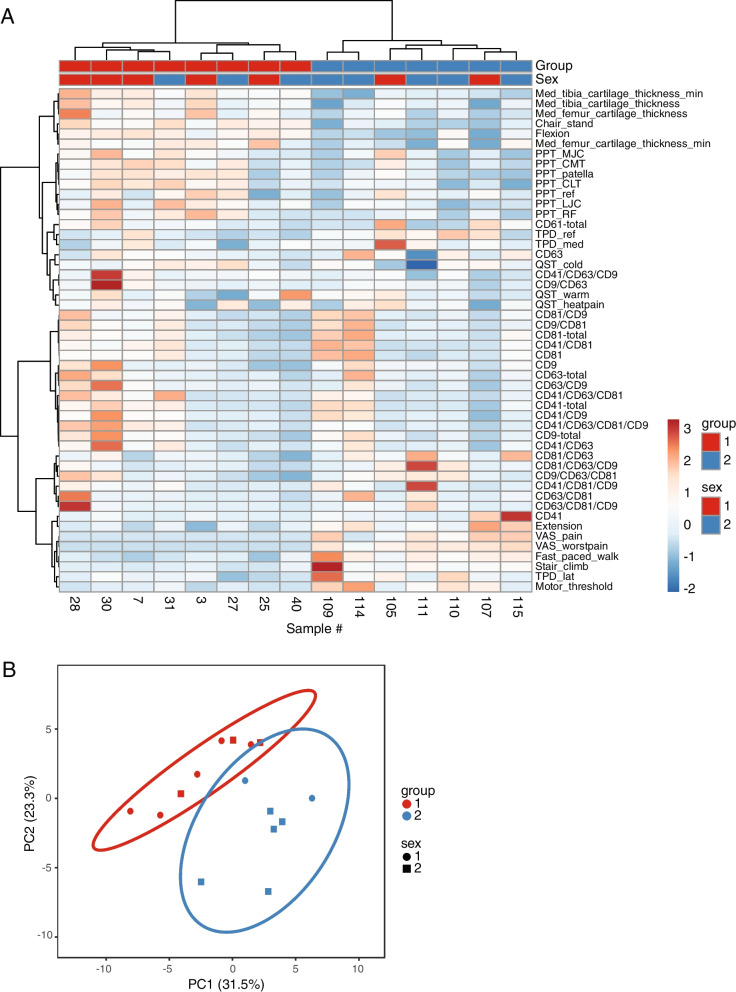
Unsupervised classification discriminates controls from knee osteoarthritis (KOA) patients. **A** Clustergram showing the *Z*-scores of variables (as indicated in rows) of the subjects (as indicated in columns), color-coded as indicated. Hierarchical clustering of the variables was performed in ClustVis (https://biit.cs.ut.ee/clustvis/) using the Ward method [52]. Distances of the bonds on the left side to the color-coded clustergram grow with the dissimilarity between variables across subjects, while the distances of the bonds shown on the top increase with the dissimilarity between subjects across the space of variables. Diagnosis group (1 = control; 2 = KOA) and sex of the subjects (1 = male; 2 = female) were not used for clustering. **B** Scatter plot showing the controls and KOA patients as single dots, projected in the space of the two major principal components (PC1–2). One KOA patient was not included in these analyses due to missing values of cartilage thickness

Groups of strongly correlated variables encompassed specific EV subpopulations and pain and function variables, indicating that EVs could have use as biomarkers for pain, disability, and progress of KOA. The existence of these correlations prompted us to ask if these variables could be predictors of clinically determined KOA. For this purpose, we conducted unsupervised HC of the subjects and variables measured from both the controls and KOA patients but excluded confounding factors (age, BMI) and LICI and Map TA with missing values. HC perfectly discriminated the control and KOA groups (Fig. 2A). The diagnostic power of the combined dataset of medial cartilage thickness, knee function, pain, sensation, and EV molecular signatures was confirmed by PCA, where the first two principal components were sufficient to distinguish subjects from the two groups, irrespective of sex (Fig. 2B). Yet, the clustering was only partially achieved when the analysis was performed using EV subpopulations alone (data not shown), indicating that the other parameters played a prominent role in the correct classification of the subjects.

We complemented the unsupervised approaches described above by a supervised RF discrimination of the subjects based on the combined dataset of medial cartilage thickness, knee function, pain, sensation, and EV molecular signatures. In agreement with HC and PCA, RF-based classification discriminated on average 91% of the controls and KOA patients, with minimum medial tibia cartilage thickness, fast-paced walk, stair-climb, and chair-stand tests, and worst VAS pain showing the highest feature importance scores (Supplementary Fig. S6). Of EV subpopulations, CD81/CD63/CD9-, CD81/CD63-, and CD41/CD63/CD9-positive sEVs had the highest feature importance scores, and, as mentioned above, the first two of these also showed statistically higher counts in KOA serum. The classification efficiency fell to 51–52%, when only EV molecular signatures were used for RF model training/testing (data not shown).

**Fig. 3:**
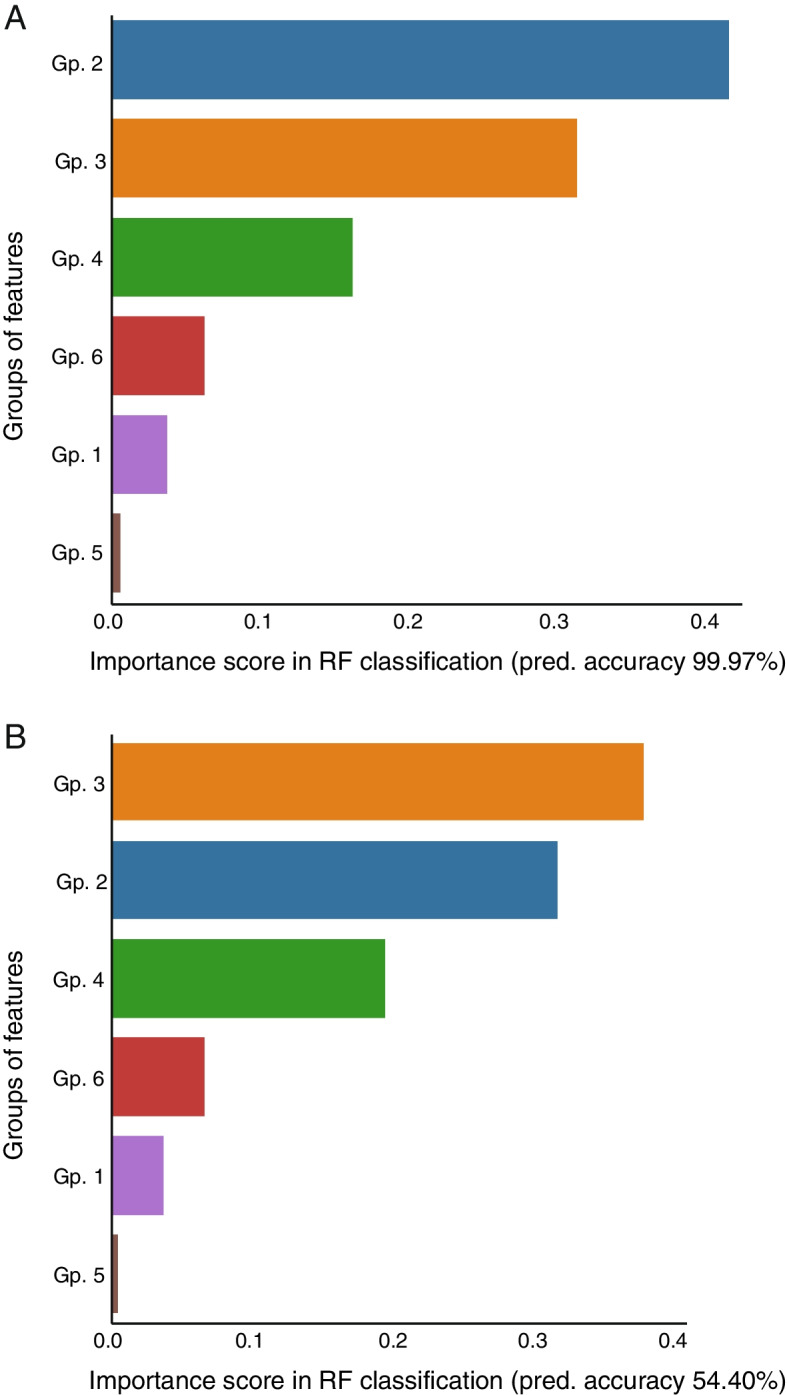
Random forest (RF)-based classification of controls and knee osteoarthritis patients. **A** Feature importance scores of each group of variables (Gp.) in the RF-based classification of the subjects, displayed as bar charts. Shown scores are averages of the groups’ importance scores over 100 RFs of 100 trees each, run on a dataset enriched 1000-fold *before* splitting training and testing data (see the “Materials and methods” section for details). **B** Feature importance scores of each Gp. in the RF-based classification of the subjects, displayed as bar charts. Shown scores are averages of the groups’ importance scores over 100 RFs of 100 trees each, run on a dataset enriched 1000-fold *after* splitting training and testing data (see the “Materials and methods” section for details)

As multicollinearity between variables might, in principle, confound the estimation of featuresʼ importance in RF classification, we sampled the variable groups (Fig. 1) to create a 1000-fold enriched dataset with only 6 features, one representing each group of variables as detailed in [54] (see also the “Materials and methods” section). This approach eliminates de facto a large fraction of the multicollinearity in the data and, at the same time, increases the size of the dataset. Data enrichment that was performed *before* splitting training and testing sets resulted in almost perfect predictions, confirming the information redundancy within each group of variables, and identified especially group 2 (6 EV subpopulations) as the most important feature in the classification (Fig. 3A). We next interrogated the power of the variable groups to predict new samples by enriching the data *after* splitting training and testing data. This approach led to poorer prognostic power, yet, the featuresʼ importance ranking was very similar to that of the control classifier (Fig. 3A), indicating that variable groups 2 (containing, e.g., CD81/CD63/CD9- and CD81/CD63-positive sEVs) and 3 (containing, e.g., fast-paced walk, stair-climb, and VAS pain variables) would be reliable classification features even for uncategorized samples (Fig. 3B). Hence, even in a dataset where multicollinearity has been strongly reduced, the counts of CD81/CD63/CD9- and CD81/CD63-positive sEVs appear as top features to distinguish KOA from the control subjects.

**Fig. 4:**
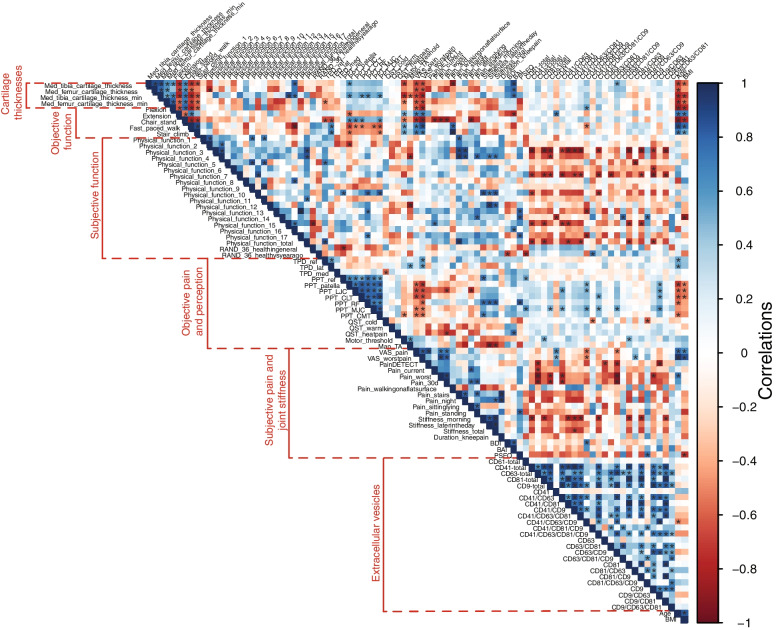
Correlogram showing the Pearson correlation coefficients between pairs of variables, computed across all subjects and categorized according to the variable groupings in Supplementary Tables S1–5. Correlation coefficients are color-coded as indicated. Dark blue (respectively red) on the correlogram indicates strong positive (respectively negative) correlations in variables’ *Z*-scores. Asterisks indicate statistically significant correlations (*p* < 0.05). Definitions of the physical function categories 1–17 are listed in Supplementary Table S3

Figure 4 represents the Pearson correlations of Fig. 1 arranged according to different variable categories (cartilage thickness, objective and subjective function and pain, EVs). Medial tibia and femur cartilage thicknesses showed inverse associations with age, BMI, and VAS pain, while their correlations to EV molecular signatures were generally weak except for the positive association between medial tibia cartilage thicknesses and counts of CD63^+^/CD9^+^ sEVs. Objective functional parameters showed negative (flexion, number of chair-stands) or positive associations (extension, duration of fast-paced walk and stair-climb tests) with age, BMI, and VAS scores. In this case, higher extension values indicate a more limited ROM. There was also a positive association between flexion and CD41^+^/CD63^+^/CD9^+^ sEVs and between extension and single-positive CD41^+^ sEVs, as well as an inverse association between the duration of fast-paced walk and CD41^+^/CD63^+^/CD9^+^ sEVs. Regarding subjective functional parameters, difficulty in performing physical activities (especially categories 3, 7, and 15 defined in Supplementary Table S3) associated inversely with several EV subpopulations. The total physical function score correlated negatively with, for instance, total counts of CD41^+^ and CD81^+^ sEVs and with CD41^+^/CD63^+^/CD81^+^ sEVs. In addition, total CD61^+^ lEVs showed an inverse association and CD41^+^/CD81^+^/CD9^+^ sEVs a positive association with the patients’ general health compared to the situation 1 year ago, according to RAND-36. In this case, higher RAND-36 scores indicate worsening of health.

Among the parameters of objective pain and sensation, tactile acuity (TPD) did not correlate with the counts of EV subpopulations, while there were positive correlations between PPTs and CD61^+^ lEVs and CD9^+^/CD63^+^, CD41^+^/CD63^+^/CD9^+^, CD41^+^/CD63^+^, and CD41^+^/CD63^+^/CD81^+^/CD9^+^ sEVs, and inverse associations between PPTs and age/BMI. rMT showed positive associations with total and single-positive CD81^+^ sEVs, while Map TA and LICI were only weakly associated with the measured EV subpopulations. With respect to subjective pain, VAS scores showed positive associations with age, BMI, and single-positive CD41^+^ sEVs, while the correlations were negative with CD41^+^/CD63^+^/CD9^+^ and CD63^+^/CD9^+^ sEVs. PainDETECT scores associated negatively with total CD63^+^ and CD9^+^ sEV counts and with, for instance, CD41^+^/CD63^+^ and CD9^+^/CD63^+^ sEVs. Stiffness parameters showed inverse correlations with many EV subpopulations, including total counts of CD41^+^ and CD81^+^ sEVs and CD41^+^/CD63^+^/CD81^+^ and CD41^+^/CD63^+^/CD81^+^/CD9^+^ sEVs. Regarding psychological well-being, BDI correlated positively with CD41^+^/CD63^+^/CD9^+^ sEV, but the associations did not reach significance for BAI. PSEQ associated negatively with BMI and positively with CD81^+^/CD63^+^/CD9^+^ sEVs.

## Discussion

To the best of our knowledge, this is the first time the associations between EV subpopulations and a wide array of variables related to cartilage erosion, physical limitations, and pain symptoms have been investigated in KOA patients. Although EVs did not exhibit clear quantitative differences between the study groups, the more detailed co-localization analysis of sEVs revealed changes in EV tetraspanin profiles. The main findings of the study were as follows: (*i*) EVs had distinct molecular signatures in control and KOA serum, (*ii*) the levels of specific EV subpopulations reflected physical limitations, joint pain, and stiffness, and (*iii*) the higher levels of CD41^+^/CD63^+^/CD9^+^ sEVs were associated with better mobility, lower functional impairment, and less pain. However, the EV tetraspanin profiles were not sufficient to distinguish between control and KOA serum in unsupervised analyses, which may partly result from the small sample size of the present study. Still, data on these EVs can provide valuable new information about how KOA symptoms are manifested at the level of cellular communication and be useful in pinpointing targets for early intervention and treatment.

**Fig. 5:**
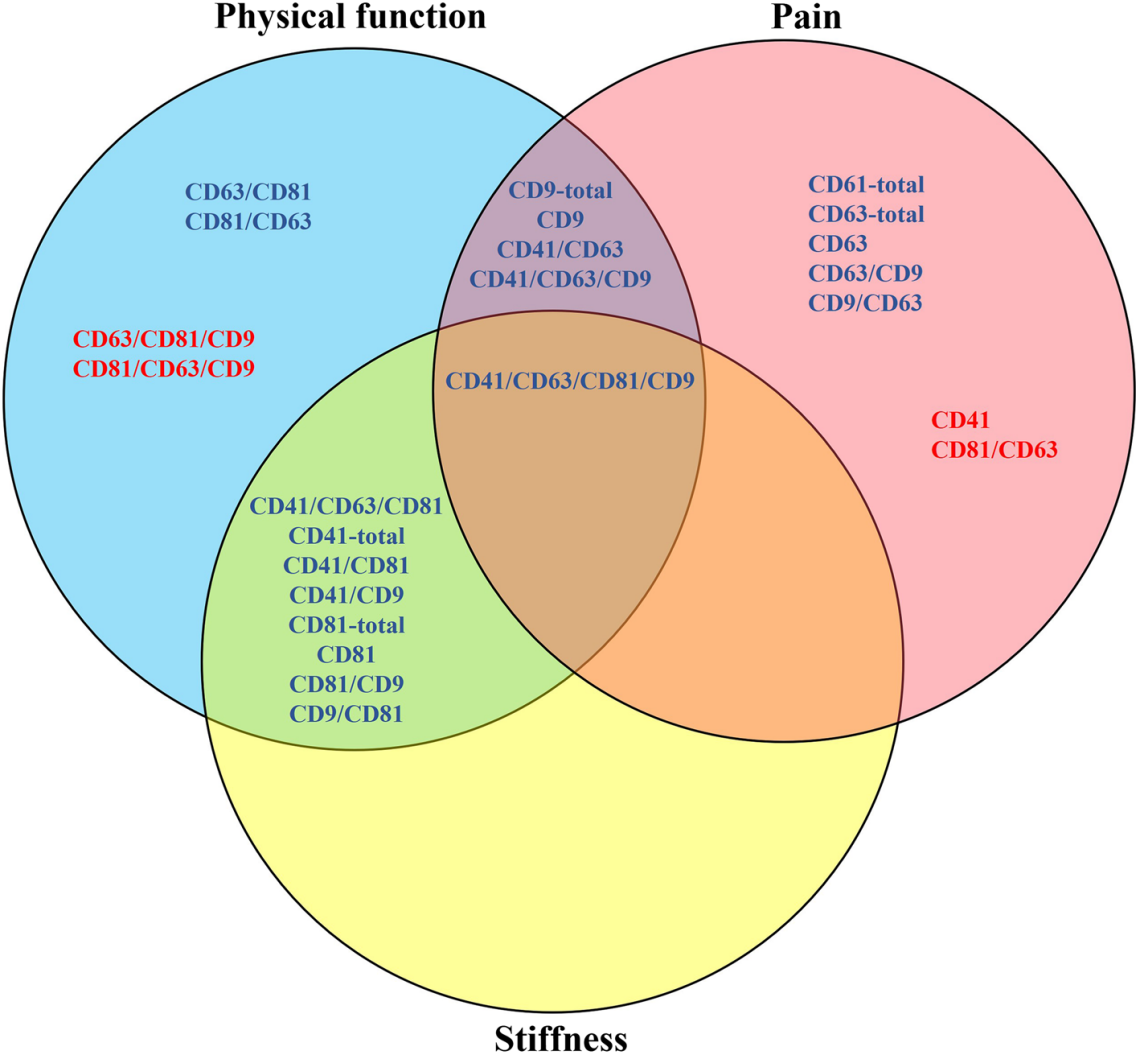
Schematic representation of the associations of selected extracellular vesicle (EV) subpopulations with different surface markers to physical performance, pain symptoms, and stiffness of the knee joint. Blue font color indicates that higher EV counts are associated with better physical function, less pain, and/or lower stiffness, whereas red font color indicates potentially aggravating factors

While the degree of articular cartilage loss showed expected associations with age, obesity, and experienced pain, it was not as clearly reflected in the serum EV parameters. Previously, CD9 or CD81 deficiency induced protective effects on articular cartilage in rodent models of arthritis [28, 29], but earlier studies on the connections of EV tetraspanins and cartilage degradation remain scarce. Regarding the associations between objective functional parameters and EV subpopulations, the ROM of the knees increased with elevated counts of CD41^+^/CD63^+^/CD9^+^ sEVs (flexion) and decreased with single-positive CD41^+^ sEVs (extension) (Fig. 5). CD41^+^/CD63^+^/CD9^+^ sEVs also increased with a better performance in the fast-paced walk test. The presence of CD41 in these EV subpopulations suggests that they were released from platelets. Regarding subjective functionality, certain self-reported physical limitations were associated with the levels of serum EV subpopulations. The strongest inverse correlations were observed for the physical function scores representing pain on movement: getting up from sitting, getting in/out of the car, and getting on/off the toilet. The total counts of CD41^+^ and CD81^+^ sEVs, and the counts of CD41^+^/CD63^+^/CD81^+^ sEVs increased with smaller physical limitations reported by the KOA patients. Previous data on the connections between EVs and knee stiffness are also scarce. In the present study, the patients with higher total counts of CD41^+^ and CD81^+^ sEVs, and higher counts of CD41^+^/CD63^+^/CD81^+^ and CD41^+^/CD63^+^/CD81^+^/CD9^+^ sEVs reported lower stiffness in their OA knees, especially in the morning. As several sEV subpopulations with CD41 and/or CD81 associated with parameters of both physical function and stiffness (Fig. 5), these EVs could be promising targets when considering future research on ameliorating factors to treat the functional symptoms of KOA, for instance, by reversing synovial fibrosis [55].

Regarding subjective pain, VAS scores showed positive associations with single-positive CD41 sEVs from platelets, while the correlations were negative with CD41^+^/CD63^+^/CD9^+^ and CD63^+^/CD9^+^ sEVs (Fig. 5). PainDETECT scores associated inversely with total CD63^+^ and CD9^+^ sEV counts. With respect to objective pain and sensation, elevated CD41^+^/CD63^+^/CD9^+^, CD41^+^/CD63^+^/CD81^+^/CD9^+^, CD41^+^/63^+^, CD61^+^, and CD9^+^/CD63^+^ EVs associated with higher PPTs. Lower levels of these EV subpopulations may, thus, be connected with local pressure hyperalgesia [34]. Previously, MSC-EVs expressing, e.g., CD9, CD63, and/or CD81 induced chondroprotective and anti-inflammatory effects and cartilage regeneration in animal models [56], but pain parameters were not always reported. As earlier studies have usually concentrated on MSC-EVs and their relations to pain, we should exert some caution when comparing these previous results to the present ones on circulatory EVs. Still, according to He et al. [13], bone marrow MSC-derived EXOs with CD63 promoted cartilage repair and extracellular matrix synthesis as well as alleviated inflammatory and neuropathic knee pain in OA rats. In Ai et al. [15], CD9^+^ MSC-EVs were suggested to reduce OA pain by direct action on peripheral sensory neurons. Moreover, bone marrow MSC-EVs with CD9 and CD63 relieved OA pain via abrogation of aberrant nerve invasion in subchondral bone [14]. These pain-relieving effects of EVs in OA animals yield support from our findings of inverse associations between CD63^+^ and CD9^+^ sEVs and different pain scores from the painDETECT questionnaire. In a rodent model of nerve injury, EXOs from umbilical cord MSCs with CD63 and CD81 inhibited neuroinflammation and neuropathic pain and promoted the expression of an anti-inflammatory cytokine and neurotrophic factors [57], and spinal cord injury increased the counts of plasma CD81^+^ EVs in a mouse model [58]. On the other hand, anti-CD81 vectors administered into the ankle joints of rats with collagen-induced arthritis suppressed joint destruction [29]. While the neurodegenerative pain mechanisms are not directly applicable to musculoskeletal pain, it seems that partly similar EV subpopulations could be involved in pain alleviation/aggravation in both cases.

Depression and anxiety are associated symptoms of chronic pain [2], and, expectedly, KOA has also been connected with deteriorated mental health [59]. In the present study, the KOA patients with elevated CD41^+^/CD63^+^/CD9^+^ sEVs reported greater BDI scores, supporting the known association between platelet activation and depression [60]. The correlations between EV subpopulations and BAI scores were nonsignificant, which suggests that while there could be an association between EVs and depressive state, the biochemistry of anxiety would either not be directly associated with EVs or it could correlate with EV subpopulations that were not measured in the present study. EV cargo, especially microRNAs, has been documented to change in circulation and cerebrospinal fluid in mental disorders [61]. Regarding the pathophysiology of major depressive disorder, EVs can associate with, for instance, neuroinflammation, neurogenesis, and depression symptoms, of which interconnectedness with depression could also be demonstrated in the present study. The observed connection between depression and CD41^+^/CD63^+^/CD9^+^ sEVs in KOA suggests that the potential of EV subpopulations as biomarkers of mental status warrants further investigation. Even though the patient population of the present study only had minimal to mild depression symptoms, EVs could relate to the well-being of patients not only with chronic OA pain but potentially also with other pain disorders or depression. However, it is necessary to study the potential link between EVs and mental health also without chronic inflammatory conditions that could per se predispose patients to depression and anxiety.

Further emphasizing the potential significance of EVs in pain perception, the measured rMTs showed positive associations with CD81^+^ sEVs, the high levels of which could reflect reduced corticospinal excitability [62]. Previously, CD9^+^ EVs from MSCs have reduced sensory neuron hyperexcitability in a mouse OA model [15]. LICI, which is a measure of GABA_B_-mediated intracortical inhibition, has been shown to increase in the primary motor cortex by chronic pain [63]. In the present study, LICI was not affected by KOA nor did it correlate with EV subpopulations. This may partly derive from the small number of measurements due to missing data and from large interindividual variation. However, it is possible that central amelioration of pain is something that could be attained by EV manipulations, and one reason for this would be the ability of peripheral EVs to cross the blood–brain barrier [61].

Study limitations to be acknowledged include the relatively small number of research subjects and the fact that all parameters were not measured from the controls. In OA studies, healthy control subjects of the same demographical group as OA patients are very difficult to recruit due to the fact that the elderly often do display joint-related symptoms or early-stage OA even when undiagnosed. Due to this, the age-matching of the patients and controls was not attained. As a result of the multi-faceted issue of overweight worsening KOA symptoms and KOA causing reduced physical exercise, it is also difficult to match KOA patientsʼ BMIs with healthy controls.

However, when the controls and KOA patients were analyzed both together and separately, there were only a few significant correlations between EV variables and age or BMI, indicating that the presence or absence of KOA and not age/BMI was the causative factor for the observed differences in the EV subpopulations between the study groups. This is supported by the relatively few significant group × age interactions (cartilage loss, flexion, walking speed, skin thermal detection, rMT, VAS pain) and group × BMI interactions (cartilage loss, flexion, skin thermal detection, reference PPT, 3 subpopulations of sEVs) observed by GLM. Many of these interactions were readily understandable as, e.g., cartilage thickness and joint mobility are affected by age. Thus, even though age and BMI did expectedly have some effects on the measured variables, it is plausible to state that KOA was the principal factor in the observed differences between experimental groups. Finally, although we identified EV markers that correlate with KOA, we have not yet explored their specificity and, thus, some other inflammatory condition could hypothetically lead to the same signature, which needs to be clarified in future studies.

To summarize, circulating EVs have emerged as a promising source of biomarkers and even therapeutic targets in KOA. Although they did not exhibit clear quantitative differences between the study groups, the more detailed co-localization analysis of sEVs revealed modifications in EV tetraspanin profiles between the controls and KOA patients. This suggests that KOA has an effect on released EVs and warrants further research on these EV subpopulations. Higher total counts of CD61^+^ lEVs and CD63^+^ sEVs could be associated with lower objective and subjective pain, respectively. Especially the sEV subpopulation with CD41/CD63/CD9 reflected several crucial aspects of general and joint health, including a higher ROM of the knee, better physical performance, and lower pain sensitivity. These findings suggest the involvement of EVs in arthritis, but further research is required to establish their roles in OA pathophysiology.

### Supplementary Information

**Additional file 1: Supplementary Figure S1.** Determination of articular cartilage thicknesses. (A) Segmented cartilage and bone labels using 3D nnU-Net, (B) geometries obtained from the segmentation after smoothing, (C) femoral cartilage thickness map with the load-bearing region illustrated, and (D) tibial cartilage thickness map with the load-bearing region illustrated, STL = stereolithography.

**Additional file 2: Supplementary Figure S2.** Gating strategy in flow cytometry analysis for large serum extracellular vesicles (EVs; 200–1000 nm). EVs were detected based on their light scatter, which was calibrated with Rosetta calibration system (Exometry, Amsterdam, the Netherlands) to set an EV diameter gate of 200–1000 nm based on side scatter (A), in which the lower limit was set to exclude noise and the upper limit to exclude cell remnants. Representative dot blots are shown for unstained (B), mouse IgG1-allophycocyanin (APC) stained (C), and mouse anti-human CD61-APC stained (D) serum EVs. The fluorescence gate was set using unstained serum sample (B), and positive events (+) were defined as events with fluorescent signal exceeding the threshold. Isotype control (C) was used to differentiate between nonspecific and specific binding of antibodies, a.u. = arbitrary unit.

**Additional file 3: Supplementary Figure S3.** Total counts of selected extracellular vesicle subpopulations with (A) CD61 determined with flow cytometry and with (B) CD41, CD63, CD81, and CD9 (mean + SE) determined with single particle interferometric reflectance imaging sensor in the serum of control and osteoarthritis (OA) patients (*n* = 8/group). There were no statistically significant differences between the groups, MIgG = isotype control.

**Additional file 4: Supplementary Figure S4.** Tetraspanin co-localization (%) in control and osteoarthritic (OA) serum (n = 8/group) analyzed by single particle interferometric reflectance imaging sensor.

**Additional file 5: Supplementary Figure S5.** Counts of selected extracellular vesicle subpopulations positive for CD41 (A), CD63 (B), CD81 (C), and CD9 (D) (mean + SE, *n* = 8/group). Asterisks denote statistically significant differences between control and osteoarthritic (OA) serum.

**Additional file 6: Supplementary Figure S6.** Random forest (RF)-based classification correctly identifies controls and knee osteoarthritis patients. Feature importance scores of each original variable in the RF-based classification of the subjects, displayed as bar charts. Shown scores are averages of the variables’ importance scores over 500 RFs of 100 trees each, run on the original dataset (16 subjects). Importance scores were computed on the data of 8 training subjects, and accuracy scores on the data of 8 testing subjects, both selected randomly at each iteration. * = significant difference from control (Mann–Whitney *U* test, Kruskal–Wallis ANOVA, *p* <0.05)

**Additional file 7: Supplementary Table S1.** Articular cartilage thicknesses in medial and lateral tibia and femur load-bearing regions of control and osteoarthritis (OA) patients (mean ± SE, *n* = 8/group). **Supplementary Table S2.** Objective functional parameters of control and osteoarthritis (OA) patients (mean ± SE, *n* = 8/group). **Supplementary Table S3.** Subjective functional parameters of osteoarthritis (OA) patients (mean ± SE, *n* = 8). **Supplementary Table S4.** Parameters of objective pain and sensation of control and osteoarthritis (OA) patients (mean ± SE, *n* = 8/group). **Supplementary Table S5.** Parameters of subjective pain, stiffness, and mental health of control and osteoarthritis (OA) patients (mean ± SE, *n* = 8/group). 

**Additional file 8: Supplementary Material S1.** Physical measurements.

**Additional file 9: Supplementary Material S2.** Image analysis for the determination of articular cartilage thickness.

**Additional file 10: Supplementary Material S3.** Random forest analysis code in Python as an annotated Jupyter notebook.

## Data Availability

All relevant data analyzed during this study are included in this published article and its supplementary information files.

## Abbreviations

APC: Allophycocyanin
BAI: Beck anxiety inventory
BDI: Beck depression inventory
BMI: Body mass index
CD: Cluster of differentiation
CL: Contralateral
EV: Extracellular vesicle
EXO: Exosome
GLM: Generalized linear model
HC: Hierarchical clustering
KOA: Knee osteoarthritis
lEV: Large extracellular vesicle
LICI: Long-interval cortical inhibition
Map TA: Map *tibialis anterior* muscle
MEP: Motor evoked potential
MRI: Magnetic resonance imaging
MSC: Mesenchymal stem cell
OA: Osteoarthritis
PCA: Principal component analysis
PPT: Pressure pain threshold
PSEQ: Pain self-efficacy questionnaire
RA: Rheumatoid arthritis
RF: Random forest
rMT: Resting motor threshold
ROM: Range of motion
RT: Room temperature
SE: Standard error
sEV: Small extracellular vesicle
SF: Synovial fluid
SP-IRIS: Single particle interferometric reflectance imaging sensor
TA: *Tibialis anterior* muscle
TMS: Transcranial magnetic stimulation
TPD: Two-point discrimination
VAS: Visual analog scale
WOMAC: Western Ontario and McMaster Universities Osteoarthritis Index
