# Supplementary material for: Tetraspanin profiles of serum extracellular vesicles reflect functional limitations and pain perception in knee osteoarthritis
Source: Arthritis Res Ther. 2024 Jan 22;26:33. doi: 10.1186/s13075-023-03234-0 (PMC10801950; doi:10.1186/s13075-023-03234-0)
Supplement: Supplementary file 1 — Additional file 1: Supplementary Figure S1. Determination of articular cartilage thicknesses. (A) Segmented cartilage and bone labels using 3D nnU-Net, (B) geometries obtained from the segmentation after smoothing, (C) femoral cartilage thickness map with the load-bearing region illustrated, and (D) tibial cartilage thickness map with the load-bearing region illustrated, STL = stereolithography. [file 13075_2023_3234_MOESM1_ESM.pdf]

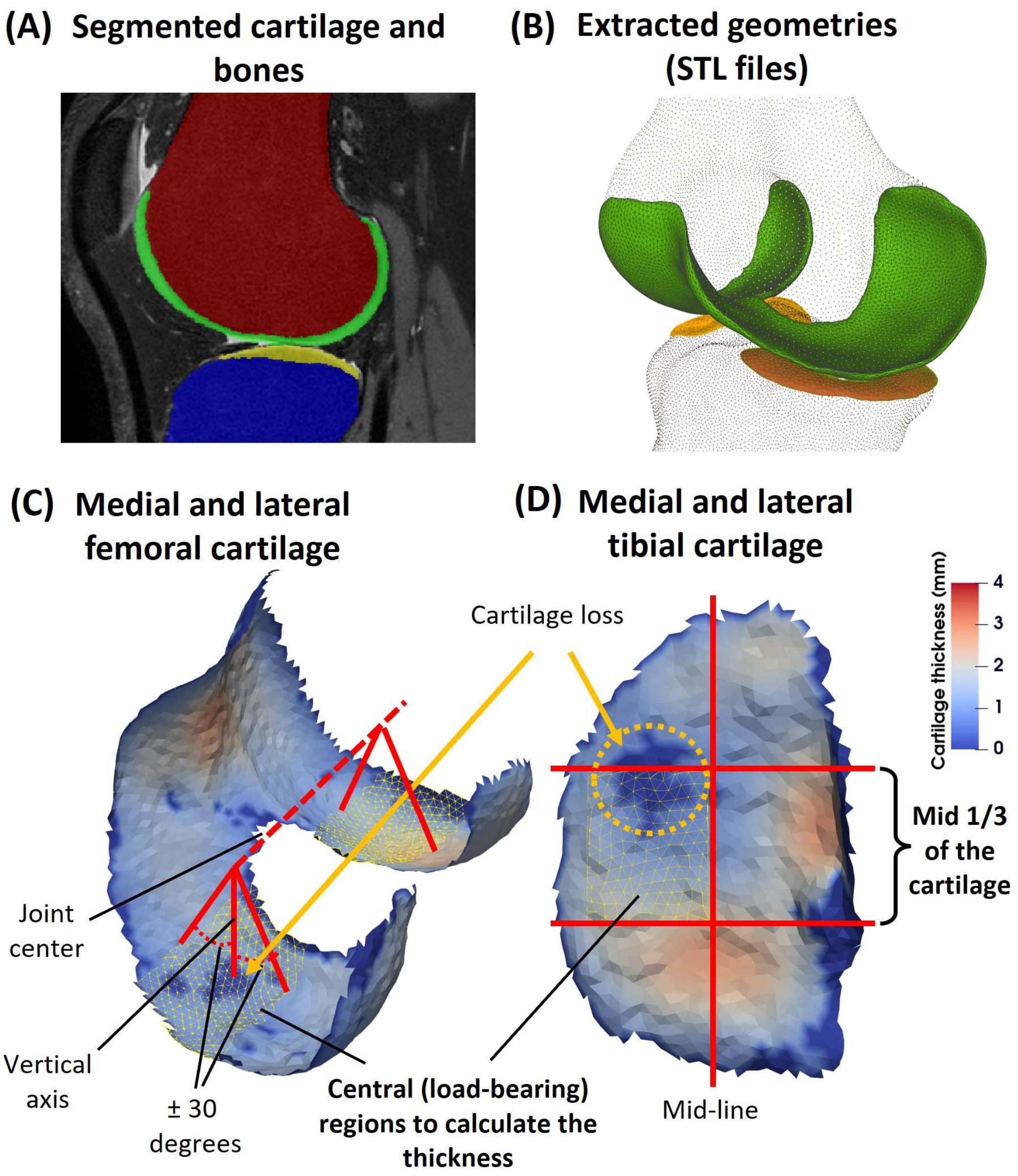

(A) Segmented cartilage and bones
(B) Extracted geometries (STL files)
(C) Medial and lateral femoral cartilage
(D) Medial and lateral tibial cartilage
Cartilage loss
Cartilage thickness (mm)
4
3
2
1
0
Mid 1/3 of the cartilage
Joint center
Vertical axis
± 30 degrees
Central (load-bearing) regions to calculate the thickness
Mid-line
