# Supplementary material for: Tetraspanin profiles of serum extracellular vesicles reflect functional limitations and pain perception in knee osteoarthritis
Source: Arthritis Res Ther. 2024 Jan 22;26:33. doi: 10.1186/s13075-023-03234-0 (PMC10801950; doi:10.1186/s13075-023-03234-0)
Supplement: Supplementary file 2 — Additional file 2: Supplementary Figure S2. Gating strategy in flow cytometry analysis for large serum extracellular vesicles (EVs; 200–1000 nm). EVs were detected based on their light scatter, which was calibrated with Rosetta calibration system (Exometry, Amsterdam, the Netherlands) to set an EV diameter gate of 200–1000 nm based on side scatter (A), in which the lower limit was set to exclude noise and the upper limit to exclude cell remnants. Representative dot blots are shown for unstained (B), mouse IgG1-allophycocyanin (APC) stained (C), and mouse anti-human CD61-APC stained (D) serum EVs. The fluorescence gate was set using unstained serum sample (B), and positive events (+) were defined as events with fluorescent signal exceeding the threshold. Isotype control (C) was used to differentiate between nonspecific and specific binding of antibodies, a.u. = arbitrary unit. [file 13075_2023_3234_MOESM2_ESM.pdf]

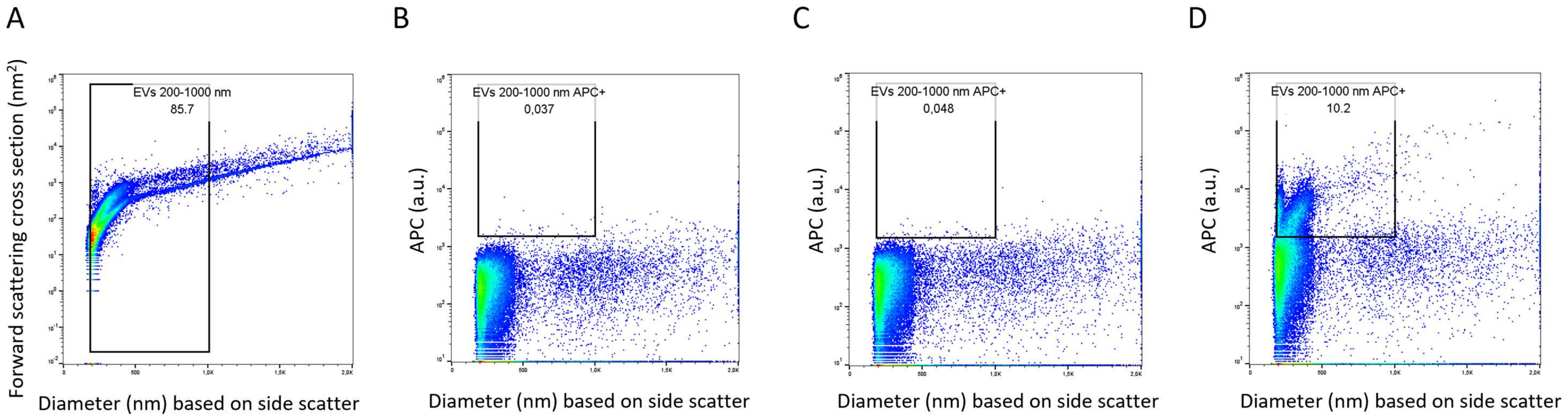
A
B
C
D
Forward scattering cross section (nm²)
EVs 200-1000 nm
85.7
Diameter (nm) based on side scatter
APC (a.u.)
EVs 200-1000 nm APC+
0,037
Diameter (nm) based on side scatter
APC (a.u.)
EVs 200-1000 nm APC+
0,048
Diameter (nm) based on side scatter
APC (a.u.)
EVs 200-1000 nm APC+
10.2
Diameter (nm) based on side scatter
