# Supplementary figures and images for: Tetraspanin profiles of serum extracellular vesicles reflect functional limitations and pain perception in knee osteoarthritis
Source: Arthritis Res Ther. 2024 Jan 22;26:33. doi: 10.1186/s13075-023-03234-0 (PMC10801950; doi:10.1186/s13075-023-03234-0)

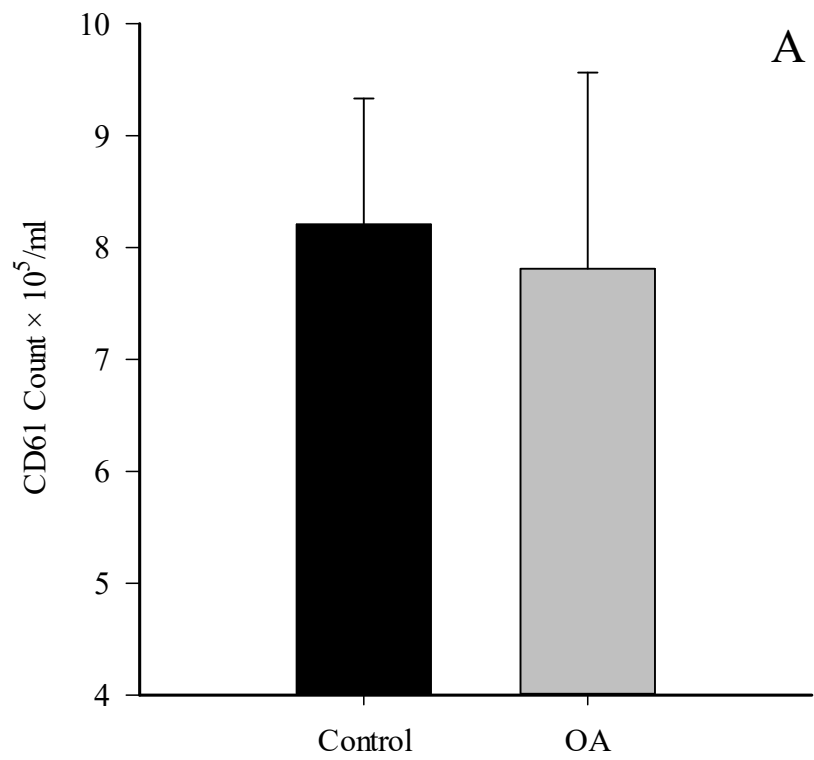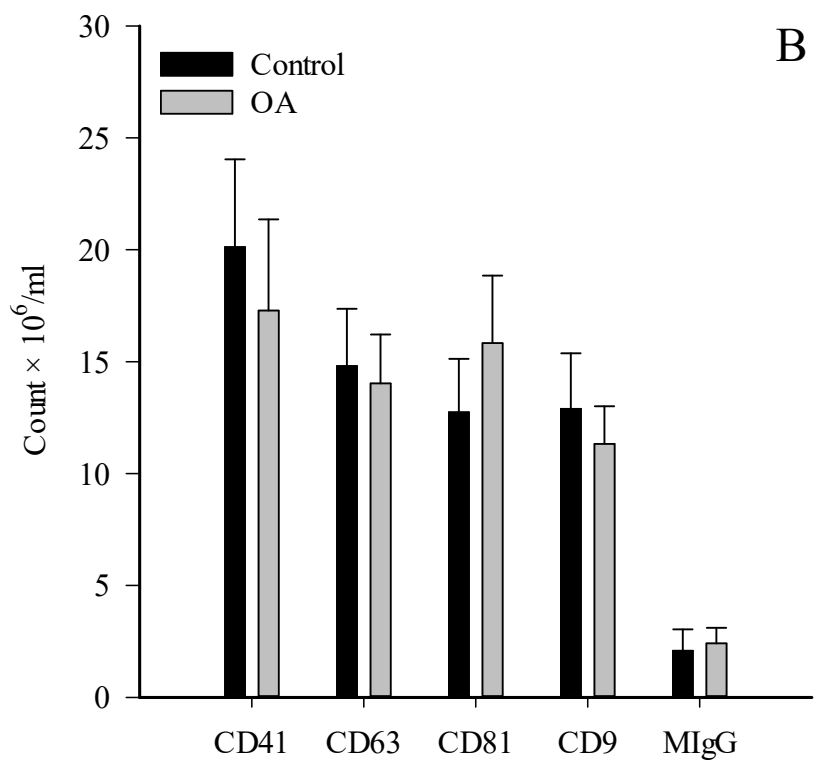

Supplement: Supplementary file 3 — Additional file 3: Supplementary Figure S3. Total counts of selected extracellular vesicle subpopulations with (A) CD61 determined with flow cytometry and with (B) CD41, CD63, CD81, and CD9 (mean + SE) determined with single particle interferometric reflectance imaging sensor in the serum of control and osteoarthritis (OA) patients (n = 8/group). There were no statistically significant differences between the groups, MIgG = isotype control. [file 13075_2023_3234_MOESM3_ESM.pdf]

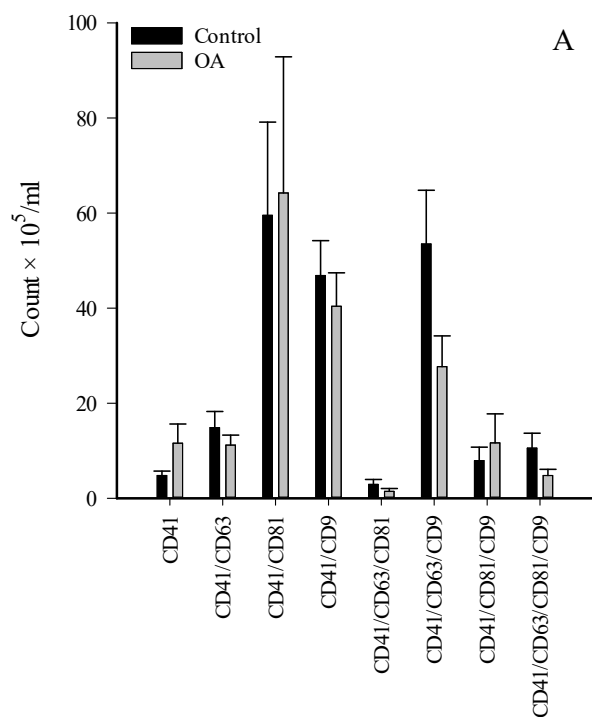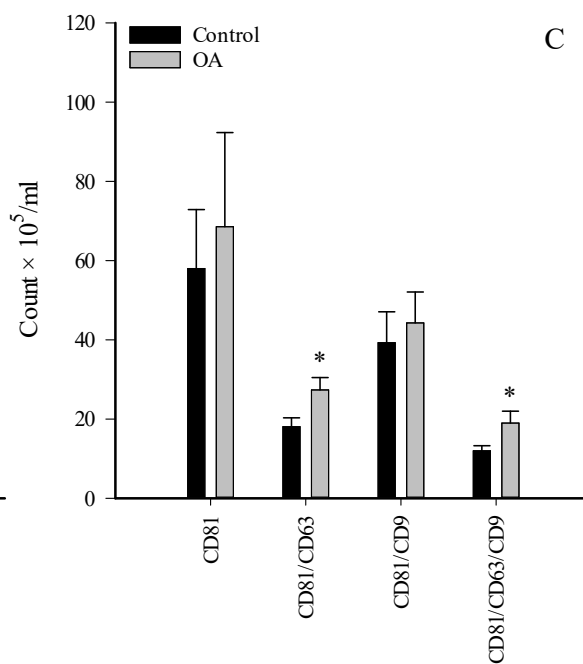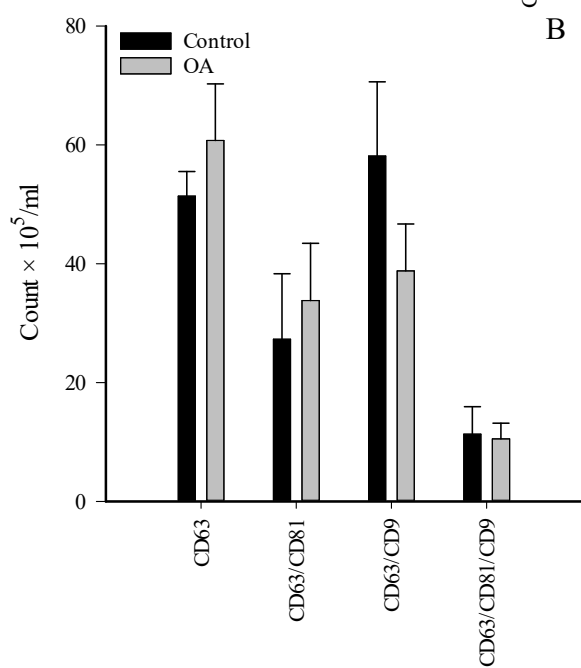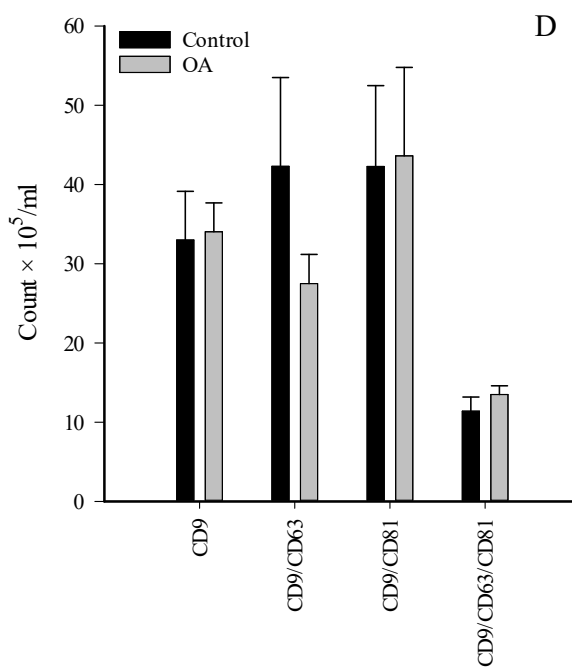

Supplement: Supplementary file 5 — Additional file 5: Supplementary Figure S5. Counts of selected extracellular vesicle subpopulations positive for CD41 (A), CD63 (B), CD81 (C), and CD9 (D) (mean + SE, n = 8/group). Asterisks denote statistically significant differences between control and osteoarthritic (OA) serum. [file 13075_2023_3234_MOESM5_ESM.pdf]

Features

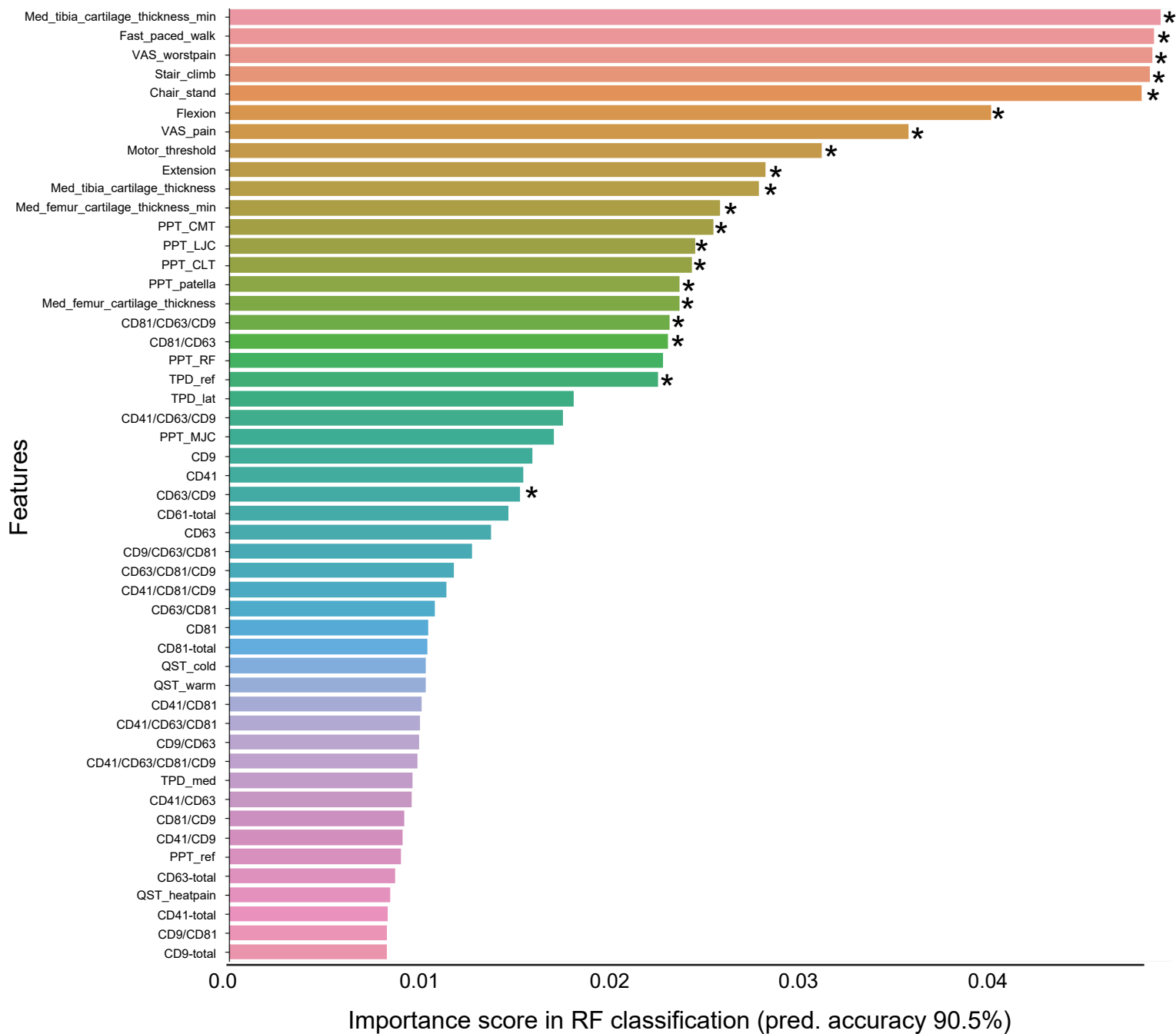

Supplement: Supplementary file 6 — Additional file 6: Supplementary Figure S6. Random forest (RF)-based classification correctly identifies controls and knee osteoarthritis patients. Feature importance scores of each original variable in the RF-based classification of the subjects, displayed as bar charts. Shown scores are averages of the variables’ importance scores over 500 RFs of 100 trees each, run on the original dataset (16 subjects). Importance scores were computed on the data of 8 training subjects, and accuracy scores on the data of 8 testing subjects, both selected randomly at each iteration. * = significant difference from control (Mann–Whitney U test, Kruskal–Wallis ANOVA, p <0.05) [file 13075_2023_3234_MOESM6_ESM.pdf]
