# Supplementary material for: Tetraspanin profiles of serum extracellular vesicles reflect functional limitations and pain perception in knee osteoarthritis
Source: Arthritis Res Ther. 2024 Jan 22;26:33. doi: 10.1186/s13075-023-03234-0 (PMC10801950; doi:10.1186/s13075-023-03234-0)
Supplement: Supplementary file 4 — Additional file 4: Supplementary Figure S4. Tetraspanin co-localization (%) in control and osteoarthritic (OA) serum (n = 8/group) analyzed by single particle interferometric reflectance imaging sensor. [file 13075_2023_3234_MOESM4_ESM.pdf]

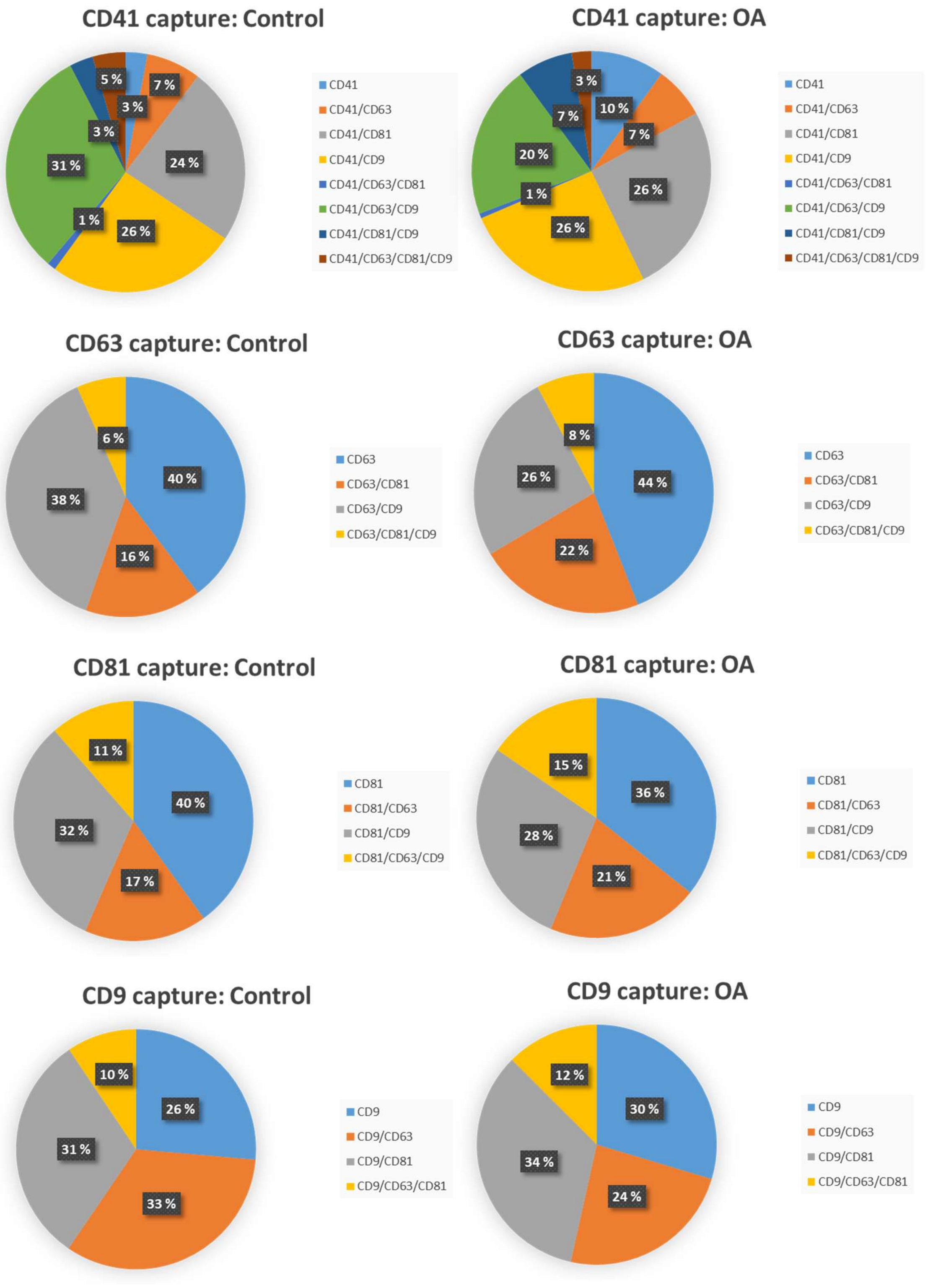
CD41 capture: Control
5 %
7 %
3 %
3 %
24 %
31 %
1 %
26 %
CD41
CD41/CD63
CD41/CD81
CD41/CD9
CD41/CD63/CD81
CD41/CD63/CD9
CD41/CD81/CD9
CD41/CD63/CD81/CD9
CD41 capture: OA
3 %
10 %
7 %
7 %
20 %
26 %
1 %
26 %
CD41
CD41/CD63
CD41/CD81
CD41/CD9
CD41/CD63/CD81
CD41/CD63/CD9
CD41/CD81/CD9
CD41/CD63/CD81/CD9
CD63 capture: Control
6 %
40 %
38 %
16 %
CD63
CD63/CD81
CD63/CD9
CD63/CD81/CD9
CD63 capture: OA
8 %
26 %
44 %
22 %
CD63
CD63/CD81
CD63/CD9
CD63/CD81/CD9
CD81 capture: Control
11 %
40 %
32 %
17 %
CD81
CD81/CD63
CD81/CD9
CD81/CD63/CD9
CD81 capture: OA
15 %
36 %
28 %
21 %
CD81
CD81/CD63
CD81/CD9
CD81/CD63/CD9
CD9 capture: Control
10 %
26 %
31 %
33 %
CD9
CD9/CD63
CD9/CD81
CD9/CD63/CD81
CD9 capture: OA
12 %
30 %
34 %
24 %
CD9
CD9/CD63
CD9/CD81
CD9/CD63/CD81
