# Supplementary material for: Tetraspanin profiles of serum extracellular vesicles reflect functional limitations and pain perception in knee osteoarthritis
Source: Arthritis Res Ther. 2024 Jan 22;26:33. doi: 10.1186/s13075-023-03234-0 (PMC10801950; doi:10.1186/s13075-023-03234-0)
Supplement: Supplementary file 7 — Additional file 7: Supplementary Table S1. Articular cartilage thicknesses in medial and lateral tibia and femur load-bearing regions of control and osteoarthritis (OA) patients (mean ± SE, n = 8/group). Supplementary Table S2. Objective functional parameters of control and osteoarthritis (OA) patients (mean ± SE, n = 8/group). Supplementary Table S3. Subjective functional parameters of osteoarthritis (OA) patients (mean ± SE, n = 8). Supplementary Table S4. Parameters of objective pain and sensation of control and osteoarthritis (OA) patients (mean ± SE, n = 8/group). Supplementary Table S5. Parameters of subjective pain, stiffness, and mental health of control and osteoarthritis (OA) patients (mean ± SE, n = 8/group). [file 13075_2023_3234_MOESM7_ESM.pdf]

Supplementary Table S1. Articular cartilage thicknesses in medial and lateral tibia and femur load-bearing regions of control and osteoarthritis (OA) patients (mean ± SE, n = 8/group).

Variable	Control	OA	CL	P^a	P^b	P^c
Med tibia thickness, mm	1.64 ± 0.142	0.99 ± 0.154*	1.22 ± 0.117	0.023	0.125	0.986
Med femur thickness, mm	1.62 ± 0.230	0.92 ± 0.136*	1.08 ± 0.117	0.040	0.098	0.951
Min med tibia thickness, mm	1.27 ± 0.097	0.56 ± 0.082*	0.65 ± 0.130*	0.001†	0.996	0.972
Min med femur thickness, mm	0.44 ± 0.040	0.21 ± 0.059*	0.39 ± 0.057	0.042	0.001	0.026
Lat tibia thickness, mm	2.04 ± 0.192	1.92 ± 0.167	1.86 ± 0.135	0.739	0.006	0.825
Lat femur thickness, mm	1.96 ± 0.273	1.52 ± 0.109	1.58 ± 0.155	0.576	0.540	0.999
Min lat tibia thickness, mm	1.38 ± 0.095	0.97 ± 0.175	1.14 ± 0.143	0.187	0.223	0.666
Min lat femur thickness, mm	0.66 ± 0.037	0.48 ± 0.074	0.56 ± 0.057	0.176	0.264	0.787

CL = contralateral, med = medial, min = minimum, lat = lateral, * = significant difference from control (Kruskal–Wallis ANOVA), † = significant difference was retained after the Benjamini–Hochberg procedure, [a] effect of group (Kruskal–Wallis ANOVA), [b] group × age interaction (generalized linear model [GLM]), [c] group × body mass index interaction (GLM)

Supplementary Table S2. Objective functional parameters of control and osteoarthritis (OA) patients (mean ± SE, n = 8/group).

Variable	Control	OA	CL	P^a	P^b	P^c
Knee flexion, °	131 ± 2	110 ± 3*	128 ± 1†	0.001#	0.019	0.027
Knee extension, °	–3 ± 1	6 ± 2*	–0.4 ± 1†	0.002#	0.343	0.231
Chair-stand test, n	20 ± 1	9 ± 1*		0.001#	0.202	0.962
Fast-paced walk test, s/40 m	21 ± 0.9	33 ± 1.9*		0.001#	0.015	0.598
Fast-paced walk test, m/s	1.97 ± 0.091	1.23 ± 0.070*		0.001#	0.002	0.062
Stair-climb test, s	8 ± 0.5	16 ± 2*		0.001#	0.086	0.980

CL = contralateral, * = significant difference between control and OA, † = significant difference between CL and OA (Mann–Whitney *U* test, Kruskal–Wallis ANOVA), # = significant differences were retained after the Benjamini–Hochberg procedure, [a] effect of group (Mann–Whitney *U* test, Kruskal–Wallis ANOVA), [b] group × age interaction (generalized linear model [GLM]), [c] group × body mass index interaction (GLM)

Supplementary Table S3. Subjective functional parameters of osteoarthritis (OA) patients (mean ± SE, n = 8).

Variable	OA
Physical function score[a], 0–4	
Ascending stairs (1)[b]	2.0 ± 0.27
Descending stairs (2)	2.6 ± 0.32
Getting up from sitting (3)	2.3 ± 0.31
Standing (4)	1.9 ± 0.35
Bending down (5)	1.8 ± 0.49
Walking on a flat surface (6)	2.3 ± 0.16
Getting in/out of the car (7)	2.3 ± 0.25
Shopping (8)	1.8 ± 0.25
Putting on socks (9)	1.8 ± 0.37
Rising from bed (10)	1.9 ± 0.35
Taking off socks (11)	1.6 ± 0.38
Lying in bed (changing/searching position) (12)	1.9 ± 0.40
In bath/shower/sauna (13)	1.3 ± 0.16
Sitting (14)	1.0 ± 0.19
Getting on/off the toilet (15)	1.5 ± 0.19
Heavy domestic duties (16)	2.0 ± 0.27
Light domestic duties (17)	1.3 ± 0.16
Total	30.9 ± 2.70
General health score[c], 1–5	3.0 ± 0.33
General health compared to one year ago[c], 1–5	3.6 ± 0.32

[a] WOMAC questionnaire, [b] the numbering of the measured physical functions used in Fig. 4, [c] RAND-36 health-related quality of life survey

Supplementary Table S4. Parameters of objective pain and sensation of control and osteoarthritis (OA) patients (mean ± SE, n = 8/group).

Variable	Control	OA	CL	P^e	P^f	P^g
Two-point discrimination test, mm						
Knee, lateral	22.4 ± 2.72	38.8 ± 5.82	25.6 ± 2.62	0.077	0.881	0.823
Knee, medial	20.5 ± 3.02	25.0 ± 3.95	22.2 ± 3.00	0.775	0.669	0.320
Reference[a]	16.3 ± 3.98	34.1 ± 4.72*		0.020	0.725	0.547
Pressure pain threshold, kg/cm^2						
Patella	7.0 ± 0.68	4.1 ± 0.51*	5.5 ± 0.67	0.017	0.469	0.171
Lateral joint capsule	8.1 ± 1.07	4.5 ± 0.69*	4.9 ± 0.77	0.039	0.912	0.353
Medial joint capsule	7.0 ± 0.94	4.6 ± 1.02	4.9 ± 0.98	0.174	0.686	0.417
Lateral tibial condyle	6.9 ± 0.76	4.1 ± 0.63*	4.6 ± 0.44	0.038	0.727	0.122
Medial tibial condyle	5.6 ± 0.81	3.0 ± 0.66*	2.8 ± 0.42*	0.026	0.531	0.349
Rectus femoris	4.9 ± 0.65	2.9 ± 0.29	3.4 ± 0.55	0.055	0.520	0.180
Reference[b]	5.1 ± 0.56	4.7 ± 0.40		0.753	0.920	0.044
Thermal detection, °C						
Warm detection	36.0 ± 0.99	35.6 ± 0.53	37.4 ± 0.78	0.198	<0.001	0.002
Cold detection	28.5 ± 0.17	28.2 ± 0.28	28.1 ± 0.30	0.649	0.226	0.001
Heat pain	44.6 ± 1.39	44.2 ± 1.34	45.3 ± 1.32	0.739	0.022	0.795
Neuromuscular measurements						
Resting motor threshold, %[c]	48.0 ± 1.84	69.9 ± 7.87*	77. 6 ± 6.70*	0.005[†]	0.043	0.978
Map *tibialis anterior*, cm^2	1.7 ± 0.39	2.9 ± 0.73	2.9 ± 0.98	0.471	0.127	0.068
Cortical long-interval inhibition, %[d]	52.4 ± 21.54	38.8 ± 31.08	160.7 ± 113.61	0.894	0.135	0.118

CL = contralateral, [a] lateral joint line of the elbow at the level of the head of radius of the non-dominant upper extremity, [b] thenar of the non-dominant upper extremity, [c] % of the maximum stimulator output, [d] ratio of the mean conditioned motor evoked potential amplitude to the mean conditioning motor evoked potential amplitude, * = significant difference from control (Mann–Whitney *U* test, Kruskal–Wallis ANOVA), [†] = significant difference was retained after the Benjamini–Hochberg procedure, [e] effect of group (Kruskal–Wallis ANOVA), [f] group × age interaction (generalized linear model [GLM]), [g] group × body mass index interaction (GLM)

Supplementary Table S5. Parameters of subjective pain, stiffness, and mental health of control and osteoarthritis (OA) patients (mean ± SE, n = 8/group).

Variable	Control	OA	CL	P^d	P^e	P^f
VAS current pain, mm	0 ± 0.0	35 ± 9.3*	6 ± 4.2†	0.004#	0.026	0.505
VAS worst pain, mm	0 ± 0.0	81 ± 6.7*	20 ± 9.6†	0.000124#	0.088	0.724
PainDETECT score[a]		12.3 ± 1.10				
Pain current, 0–10		5.0 ± 0.91				
Pain worst		8.0 ± 0.46				
Pain 30 d		6.3 ± 0.59				
Pain walking on a flat surface[b], 0–4		2.1 ± 0.14				
Pain using stairs		2.4 ± 0.20				
Pain at night		1.7 ± 0.42				
Pain sitting or lying		1.4 ± 0.20				
Pain standing upright		1.7 ± 0.29				
Stiffness in the morning		2.4 ± 0.26				
Stiffness later in the day		2.1 ± 0.30				
Stiffness total		4.5 ± 0.53				
Duration of knee pain, years		7.1 ± 2.07				
Beck depression inventory score		4.6 ± 1.72				
Beck anxiety inventory score		28.0 ± 2.04				
Pain self-efficacy score[c]		40.3 ± 2.40				

CL = contralateral, [a] painDETECT questionnaire, [b] WOMAC questionnaire, [c] Pain self-efficacy questionnaire, * = significant difference between control and OA, † = significant difference between CL and OA (Kruskal–Wallis ANOVA), # = significant differences were retained after the Benjamini–Hochberg procedure, [d] effect of group (Kruskal–Wallis ANOVA), [e] group × age interaction (generalized linear model [GLM]), [f] group × body mass index interaction (GLM)
