# Supplementary material for: Tetraspanin profiles of serum extracellular vesicles reflect functional limitations and pain perception in knee osteoarthritis
Source: Arthritis Res Ther. 2024 Jan 22;26:33. doi: 10.1186/s13075-023-03234-0 (PMC10801950; doi:10.1186/s13075-023-03234-0)
Supplement: Supplementary file 8 — Additional file 8: Supplementary Material S1. Physical measurements. [file 13075_2023_3234_MOESM8_ESM.docx]

**Supplementary Material S1.** Physical measurements

*Visual analog scale (VAS)*

For the assessment of the severity of pain in each knee, VAS was used. Patients were asked to describe pain currently and at its worst on the VAS ranging from 0–100 mm, 0 meaning “no pain” and 100 meaning “very strong pain”.

*Range of motion*

The range of motion (flexion, extension) of each knee joint was measured with standard goniometry, while the patients were lying in the supine position [1].

*Two-point discrimination (TPD)*

Mechanical sliding calipers (baseline aesthesiometers) were used to evaluate TPD in the vertical direction on both knees. The test protocol described by Stanton *et al.* and Moberg [2, 3] was utilized. The subjects were lying down on their back eyes closed. The calipers were placed perpendicular to the skin and the subjects were asked to say “one” if they felt one point and “two” if they felt two points. At each location, an ascending and a descending run were completed (using 5 mm increments), and the average of these two runs was calculated as the TPD threshold. This resulted in a total of two TPD threshold measurements per knee. The test was performed 2 cm lateral and medial of the borders of the patella around the tibiofemoral joint line. The side (left/right), location (medial/lateral), and sequence (ascending/descending) were randomized. The reference measurement was performed on the lateral joint line of the non-dominant elbow (humeroradial joint).

*Pressure pain threshold (PPT)*

A hand-held digital pressure algometer (SAUTER GmbH, Balingen, Germany) with a 1 cm^2^ probe was used as described in Jakorinne *et al.* [4]. The probe was held perpendicular to the skin and pressure was applied at a steady rate of approximately 0.5 kg/s until the participant felt the very first sensation of pain. PPT measurements were made once at six test sites around both knees (center of patella, lateral joint capsule, lateral tibial condyle, medial joint capsule, medial tibial condyle, and *rectus femoris* muscle) and one control site on the non-dominant thenar. The first side to be tested and the order of the testing areas were randomized for each participant.

*Physical function*

Physical function was measured by using OARSI-recommended performance-based tests [5]. Prior to performance, the subjects were familiarized with the test procedures. The tests used were a 30-second chair-stand test, a 4 × 10 m fast-paced walk test, and a 12-step stair-climb test.

Standardized instructions [5]:

*From the sitting position in the middle of seat with feet shoulder width apart, flat on the floor, arms crossed at chest, stand completely up, then sit completely back down, repeatedly for 30 s. Chair should be against a wall. Count the total number of complete chair stands (up and down represents one stand) of one trial. If a full stand is completed at 30 s then this is counted in the total. Same chair is needed for re-testing*

*Walk as quickly but as safely as possible to a mark 10 m away, return, and repeat for a total distance of 40 m. Regular walking aid is allowed and recorded. Time of one trial, with turn time excluded, is recorded and expressed as speed m/s by dividing distance (40 m) by time (s)*

*Ascend and descend flight of 12 stairs as quickly as safe and comfortable. One handrail allowed but encouraged to only use legs. Total time to ascend and descend steps for one trial is recorded to nearest 100^th^ second*

**References**

1. Liikavainio T, Lyytinen T, Tyrväinen E, Sipilä S, Arokoski JP. Physical function and properties of quadriceps femoris muscle in men with knee osteoarthritis. Arch Phys Med Rehabil. 2008;89:2185–94.

2. Stanton TR, Lin C-WC, Bray H, Smeets RJEM, Taylor D, Law RYW, Moseley GL. Tactile acuity is disrupted in osteoarthritis but is unrelated to disruptions in motor imagery performance. Rheumatology. 2013;52:1509–19.

3. Moberg E. Two-point discrimination test. A valuable part of hand surgical rehabilitation, e.g. in tetraplegia. Scand J Rehabil Med. 1990;22:127–34.

4. Jakorinne P, Haanpää M, Arokoski J. Reliability of pressure pain, vibration detection, and tactile detection threshold measurements in lower extremities in subjects with knee osteoarthritis and healthy controls. Scand J Rheumatol. 2018;47:491–500.

5. Dobson F, Hinman RS, Roos EM, Abbott JH, Stratford P, Davis AM, Buchbinder R, Snyder-Mackler L, Henrotin Y, Thumboo J, et al. OARSI recommended performance-based tests to assess physical function in people diagnosed with hip or knee osteoarthritis. Osteoarthritis Cartilage. 2013;21:1042–52.
