# Supplementary material for: Tetraspanin profiles of serum extracellular vesicles reflect functional limitations and pain perception in knee osteoarthritis
Source: Arthritis Res Ther. 2024 Jan 22;26:33. doi: 10.1186/s13075-023-03234-0 (PMC10801950; doi:10.1186/s13075-023-03234-0)
Supplement: Supplementary file 9 — Additional file 9: Supplementary Material S2. Image analysis for the determination of articular cartilage thickness. [file 13075_2023_3234_MOESM9_ESM.docx]

**Supplementary Material S2.** Image analysis for the determination of articular cartilage thickness

To enhance the reliability and repeatability, the cartilage thicknesses were calculated through a fully automated pipeline implemented in Python based on the tissue geometries obtained from a deep-learning segmentation tool, nnU-Net [1, 2]. Both 2D and 3D full-resolution networks were trained using the ZIB dataset, consisting of double-echo steady-state knee MRIs and their respective segmentation labels from 507 subjects [3]. The MRIs had a slice thickness of 0.70 mm in the sagittal plane and an in-plane resolution of 0.37 × 0.37 mm. The segmentation labels consisted of femoral and tibial bones and cartilages. The 2D and 3D networks were trained using five-fold cross-validation on an NVIDIA RTX A6000 graphics processing unit. Following the training, the best configuration was determined according to the mean foreground Dice coefficient computed via cross-validation on the training dataset [2]. The ultimate mean Dice similarity coefficients were 0.986, 0.987, 0.898, and 0.866 for the femoral bone, tibial bone, femoral cartilage, and tibial cartilage, respectively.

The trained network was used to segment the MRIs of the study participants (Supplementary Fig. S1A). Next, tissue geometries in the stereolithography format were reconstructed using the marching cubes algorithm [4] followed by the Taubin filter [5] in MeshLab [6] to smooth the surfaces while affecting the tissue volume only minimally [5] (Supplementary Fig. S1B). Subsequently, the geometries were re-meshed in MeshLab [6, 7] with a 0.5 mm element size (Supplementary Fig. S1B). Finally, cartilage thicknesses were obtained by casting rays from the bone surface toward the corresponding cartilage geometry. For this, the Visualization Toolkit (VTK *v*9.2.2) ray-casting algorithm was used [8]. For every triangle in the bone mesh, a ray was cast in the direction of the surface normal (outwards). Thereafter, the last intersection of the ray with the cartilage surface, if it existed, was considered the contact surface of the cartilage, and the length of the ray between the bone surface and the intersection point was considered cartilage thickness at that triangle. Zero cartilage thickness was assigned to the bones’ triangle in the manner that its ray had no intersections with the corresponding cartilage mesh.

It was a challenge to detect regions with total cartilage loss from the surrounding bone, as both regions have zero cartilage thickness (see Supplementary Fig. S1C–D). To overcome this challenge, first, we obtained an initial estimate for the geometry of the subjects’ cartilages, *i.e.*, what the geometry of the cartilages would have been in the healthy state. To this end, we adopted segmented knee cartilages of a healthy subject as the template [9]. Next, we used the template’s and study subjects’ MRIs and the 12 degrees of freedom (non-rigid) affine registration tool [10] to scale the template to each study subject. To enhance the accuracy, registration and scaling were performed separately for the femoral cartilage and lateral and medial tibial cartilages. Lastly, the subjects’ cartilage region (Supplementary Fig. S1C–D), including regions with total cartilage loss, was considered as the sum of the region obtained from the scaled template (thickness set to 0) and the region obtained from auto-segmentation (thickness >0, explained in the previous paragraph).

Finally, the cartilage thickness was extracted from the load-bearing regions of the medial and lateral femoral and tibial cartilages [1]. Adopted from our previous studies [1, 9], femoral cartilage was first divided into the medial and lateral regions. Then, the medial and lateral femoral regions were each subdivided into anterior, central, and posterior regions (Supplementary Fig. S1C). The medial and lateral tibial cartilages were each subdivided into six regions, *i.e.*, three anteroposterior (anterior, central, and posterior) and two mediolateral (medial and lateral) regions (Supplementary Fig. S1D). The central regions of the cartilages were considered the load-bearing region (Supplementary Fig. S1C–D). From these three selected regions, we calculated the mean cartilage thickness (mean of all values) and the minimum cartilage thickness. To calculate the minimum thickness, the triangle with the minimum thickness and its neighboring triangles were first located. The minimum thickness was then calculated as the mean value of the selected triangles.

**References**

1. Esrafilian A, Chandra SS, Gatti AA, Nissi M, Mustonen A-M, Säisänen L, Reijonen J, Nieminen P, Julkunen P, Töyräs J, et al. An automated and robust tool for musculoskeletal and finite element modeling of the knee joint. bioRxiv. 2023, doi: https://doi.org/10.1101/2023.10.14.562320.

2. Isensee F, Jaeger PF, Kohl SAA, Petersen J, Maier-Hein KH. nnU-Net: A self-configuring method for deep learning-based biomedical image segmentation. Nat Methods. 2021;18:203–11.

3. Ambellan F, Tack A, Ehlke M, Zachow S. Automated segmentation of knee bone and cartilage combining statistical shape knowledge and convolutional neural networks: Data from the Osteoarthritis Initiative. Med Image Anal. 2019;52:109–18.

4. Lorensen WE, Cline HE. Marching cubes: A high resolution 3D surface construction algorithm. SIGGRAPH ´87: Proceedings of the 14th Annual Conference on Computer Graphics and Interactive Techniques, 1987, 163–9.

5. Taubin G. A signal processing approach to fair surface design. SIGGRAPH ´95: Proceedings of the 22nd Annual Conference on Computer Graphics and Interactive Techniques, 1995, 351–8.

6. Cignoni P, Callieri M, Corsini M, Dellepiane M, Ganovelli F, Ranzuglia G. MeshLab: An open-source mesh processing tool. Eurographics Italian Chapter Conference, Scarano V, De Chiara R, Erra U, Eds. The Eurographics Association, 2008, 129–36.

7. Hoppe H, DeRose T, Duchamp T, McDonald J, Stuetzle W. Mesh optimization. SIGGRAPH ´93: Proceedings of the 20th Annual Conference on Computer Graphics and Interactive Techniques, 1993, 19–26.

8. Schroeder W, Martin K, Lorensen B. The visualization toolkit: An object-oriented approach to 3D graphics, 4th ed. Kitware, Clifton Park, NY, 2006.

9. Esrafilian A, Stenroth L, Mononen ME, Vartiainen P, Tanska P, Karjalainen PA, Suomalainen J-S, Arokoski JPA, Saxby DJ, Lloyd DG, et al. Toward tailored rehabilitation by implementation of a novel musculoskeletal finite element analysis pipeline. IEEE Trans Neural Syst Rehabil Eng. 2022;30:789–802.

10. Modat M, Cash DM, Daga P, Winston GP, Duncan JS, Ourselin S. Global image registration using a symmetric block-matching approach. J Med Imaging. 2014;1:024003.
